# Multilevel Mechanisms and Clinical Efficacy of Psilocybin in Depression Treatment: A Systematic Review

**DOI:** 10.3390/ijms27188052

**Published:** 2026-09-10

**Authors:** Xizhen Zhang, Xiaowen Zhang, Haiyu Zhang, Sheng Wei

**Affiliations:** 1 Institute for Chinese Medicine and Brain Science, Shandong University of Traditional Chinese Medicine, Ji’nan 250014, China; 2024111580@sdutcm.edu.cn; 2 School of Health, Shandong University of Traditional Chinese Medicine, Ji’nan 250014, China; 19707001173@163.com (X.Z.); 19511585106@163.com (H.Z.)

**Keywords:** psychedelics, psilocybin, psilocin, depression, serotonin, escitalopram

## Abstract

Depression, characterized by persistent low mood and anhedonia, represents a prevalent affective disorder imposing substantial medical and economic burdens. Current first-line antidepressants are limited by delayed onset and treatment resistance. Psilocybin (PSI) has re-emerged as a promising therapeutic agent with rapid, sustained antidepressant effects. This systematic review was conducted following the Preferred Reporting Items for Systematic Reviews and Meta-Analyses (PRISMA) 2020 guidelines and prospectively registered with International Prospective Register of Systematic Reviews (PROSPERO) (CRD420261425626). Comprehensive searches of PubMed, Web of Science, and ScienceDirect identified studies published between January 2021 and March 2026 that examined the antidepressant mechanisms and clinical efficacy of PSI. Of 949 records screened, 46 original studies were included. We synthesized evidence across the molecular, cellular, neural circuit, and clinical dimensions. Our findings suggest that psilocin, the active metabolite, may exert antidepressant effects through multilevel mechanisms, including 5-hydroxytryptamine receptor 2A (5-HT_2a_) receptor activation, brain-derived neurotrophic factor (BDNF)-tropomyosin receptor kinase B(TrkB) signaling, hippocampal neurogenesis, and default mode network functional connectivity remodeling. Clinical trials confirm that single or limited-dose PSI (particularly 25 mg) rapidly and sustainably alleviates symptoms in treatment-resistant depression, with efficacy comparable or superior to that of escitalopram. This multilevel framework bridges mechanistic insights with clinical translation, supporting PSI as a novel therapeutic pathway for depression.

## 1. Introduction

Depression constitutes an affective disorder arising from chronic negative emotional accumulation, manifesting clinically as sustained low mood, loss of interest or pleasure, and pronounced fatigue, with severe cases potentially involving suicidal ideation; its severity spectrum ranges from mild to debilitating, significantly compromising daily functioning and occupational capacity while threatening physical and psychological well-being [1]. Recent epidemiological surveys indicate that depression affects approximately 3.8% of the global population, with higher prevalence among adult females [2], and is projected to become the second most common disease worldwide [3]. Current therapeutic strategies predominantly rely on pharmacological intervention, with monoaminergic agents—including SSRIs (e.g., escitalopram, paroxetine, sertraline), TCAs (e.g., amitriptyline, amoxapine), and SNRIs (serotonin–norepinephrine reuptake inhibitors; e.g., venlafaxine, duloxetine)—serving as first-line treatments. Despite their widespread adoption due to moderate efficacy, low addictive potential, and favorable safety profiles, a significant proportion of patients experience a delayed onset of action and discontinuation syndromes with these agents [4,5]. Furthermore, MDD, a common clinical phenotype of depression, exhibits treatment resistance in approximately 10–30% of patients receiving standard interventions [6]. Recent evidence indicates that roughly 27% of patients with MDD present with elevated levels of high-sensitivity C-reactive protein (hs-CRP ≥ 3 mg/L) and demonstrate poor response to monoaminergic antidepressants [7], suggesting that conventional pharmacotherapy fails to address specific inflammatory endophenotypes within the treatment-resistant subpopulation.

Psychedelics comprise a class of naturally occurring or synthetic compounds that act on the central nervous system to induce alterations in sensory perception, emotional states, and psychological processes, accompanied by distortions in temporal and spatial perception, illusions, or hallucinations [8]. Based on their agonistic activity at the 5-HT_2a_ receptor, psychedelics are classified as classical (e.g., PSI, lysergic acid diethylamide [LSD]) or non-classical (e.g., ketamine, ibogaine) [9]. Following widespread abuse and sociopolitical stigmatization during the 1960s, the U.S. Controlled Substances Act placed these compounds under the most stringent regulatory schedules, with parallel international restrictions [10], relegating psychedelic research to the periphery of psychiatric inquiry [11]. However, advances in neuroscience and the persistent challenge of treatment-resistant psychiatric conditions have repositioned psychedelics at the forefront of neuroscientific investigation. These compounds demonstrate anti-inflammatory effects through reducing interleukin-6 (IL-6) and tumor necrosis factor-α (TNF-α) levels, modulation of neuroplasticity, and attenuation of default mode network (DMN) activity [9,12]. Specifically, psilocin binds to tropomyosin receptor kinase B (TrkB), increasing dendritic spine density and promoting endogenous brain-derived neurotrophic factor (BDNF) signaling to exert antidepressant effects [9,12]. Among classical psychedelics, PSI—a naturally occurring hallucinogenic compound synthesized by psychedelic mushrooms [13]—is found in nearly 200 psychoactive Psilocybe species [14,15]. Pharmacologically, PSI is a prodrug with negligible activity of its own: following oral administration, it is rapidly and almost completely dephosphorylated by alkaline phosphatases and non-specific esterases in the intestine, liver, and kidneys such that virtually no intact PSI is detectable in human plasma [16,17]. The resulting psilocin (4-hydroxy-*N*,*N*-dimethyltryptamine) constitutes the active moiety mediating the acute psychedelic effects and therapeutic actions of psilocybin; it is absorbed with a Tmax of approximately 1.8–4 h [18], exhibits linear pharmacokinetics across the clinically relevant dose range, and displays an elimination half-life of approximately 3 h [17]. Accordingly, throughout this review, mechanistic and clinical effects are attributed to psilocin, whereas “PSI” denotes the administered prodrug. The favorable safety profile and demonstrated efficacy in patients requiring rapid therapeutic response and those with TRD establish it as a compelling treatment alternative [19,20]. Although structurally homologous to serotonin (5-HT), psilocin—the active metabolite of PSI—exhibits greater binding affinity for 5-HT receptors, constituting the critical molecular basis for its psychoactive effects, including antidepressant and anxiolytic properties [15]. Additionally, microdosing studies reveal improvements in social connectedness, emotional resilience, and interpersonal relationship quality [21], demonstrating broad-spectrum psychoregulatory effects extending beyond mere mood enhancement. Nevertheless, current PSI research faces several bottlenecks: the neurobiological mechanisms underlying psilocin’s antidepressant effects remain only partially elucidated, significant phenotypic gaps exist between animal behavioral paradigms and core symptom dimensions of human depression, and interspecies pharmacokinetic differences severely constrain translational progress from mechanistic discovery to clinical application.

Accordingly, this systematic review distinguishes itself from previous fragmented mechanistic reports by constructing a multilevel integrative framework spanning from molecular targets to clinical outcomes. We examine psilocin’s binding affinity for 5-HT_2a_ receptors and TrkB, downstream signaling pathways, dendritic spine remodeling, synaptic plasticity enhancement and neurogenesis, and functional connectivity modulation within the DMN, corticolimbic circuits, and key brain regions. By correlating mechanistic evidence with clinical efficacy, we aim to provide a multilevel evidence framework for PSI-based depression therapy and theoretical insights for next-generation rapid-acting antidepressant drug development.

## 2. Methods

### 2.1. Inclusion Criteria

This review included original research encompassing randomized controlled trials, non-randomized controlled trials, cohort studies, case–control studies, animal experiments, and cellular/molecular mechanistic studies. All included studies employed PSI as the intervention or research target, with content addressing at least one of the following dimensions: molecular mechanisms (including but not limited to the 5-HT_2a_ receptor, TrkB, and BDNF signaling pathways), cellular mechanisms (including but not limited to dendritic spine remodeling, neurogenesis, and glial cell alterations), neural circuits (including but not limited to the DMN and corticolimbic connectivity), or clinical efficacy. Publications were restricted to those published in English between January 2021 and March 2026, Furthermore, included studies were required to demonstrate sound experimental design, rigorous data collection and analytical methods, and reliable and reproducible results.

### 2.2. Exclusion Criteria

We excluded the following publication types: reviews, systematic reviews, meta-analyses, grey literature, letters, commentaries, case reports, conference abstracts, editorials, guidelines, and consensus statements (non-original research). Studies unrelated to depression (e.g., exclusively investigating anxiety, addiction, or end-of-life anxiety, without explicit depression-related outcome reporting) or unrelated to PSI (e.g., investigating other psychedelics or non-psychedelic interventions) were excluded. Furthermore, studies lacking relevance to molecular, cellular, neural circuit, or clinical efficacy dimensions (e.g., solely describing pharmacological safety, pharmacokinetics, or adverse-effect profiles) were also excluded. Duplicate publications across multiple databases or formats were eliminated; non-English publications were excluded.

### 2.3. Search Strategy

This systematic review was conducted and reported in accordance with the Preferred Reporting Items for Systematic Reviews and Meta-Analyses (PRISMA) guidelines and prospectively registered with PROSPERO (CRD420261425626). Comprehensive literature searches were performed across the PubMed, Web of Science, and ScienceDirect databases to identify relevant studies published between January 2021 and March 2026. This timeframe was selected to capture the most current and methodologically robust evidence in this rapidly evolving field. The period begins with the landmark 2021 COMP001 trial, the first large-scale double-blind RCT of psilocybin for treatment-resistant depression, which marked a turning point and spurred a surge of subsequent mechanistic and translational studies. Consequently, older reports, often constrained by small samples and less rigorous designs, were excluded. The search strategy incorporated the following terms: (“Psilocybin” OR “Psilocybine” OR “Psilocibin” OR “Psilocybe” OR “Psilocin”) AND (“Depression” OR “Depressive Symptom” OR “Emotional Depression”).

**Figure 1:**
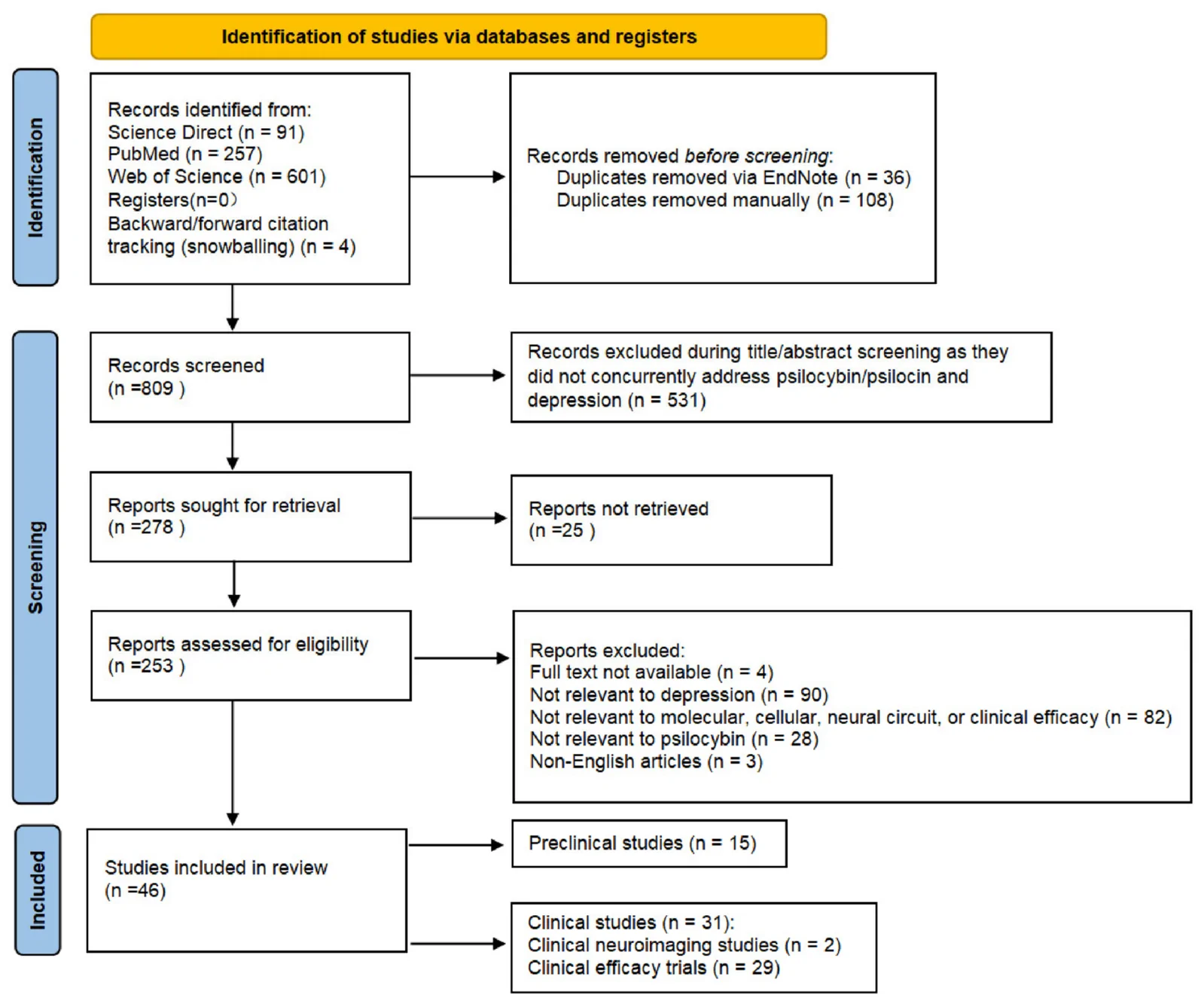
PRISMA flow diagram demonstrating study screening, evaluation, and final inclusion process.

To complement these electronic database searches, we performed backward and forward citation tracking (snowballing) on the reference lists of all initially included studies. The first round of snowballing, applied to the 42 studies retrieved from database searches, identified four additional eligible studies. A second round of snowballing on these four newly identified studies yielded no further records, resulting in a final inclusion of 46 original studies. The study selection process, including screening, eligibility assessment, and final inclusion, is summarized in the PRISMA flow diagram (Figure 1).

### 2.4. Sensitivity Validation of the Search Strategy

To assess whether the constructed search syntax inadvertently excluded pertinent records due to Boolean logic errors or insufficient term coverage, we performed a sensitivity check using a representative benchmark set. Given that database indexing coverage varies across platforms, we selected a subset of highly representative studies (*n* = 10) from our preliminary scoping search—covering randomized controlled trials, neuroimaging investigations, and preclinical mechanistic reports—that were confirmed to be present in each database (PubMed, Web of Science, and ScienceDirect). We then formally tested whether our final search string could successfully retrieve these benchmark records within their host databases. The query demonstrated high sensitivity, successfully capturing all 10 selected reference studies across the three platforms. This verification confirms that the core Boolean syntax and keyword combination were sufficiently robust and that no critical studies were systematically excluded due to search-strategy limitations.

### 2.5. Data Extraction and Analysis

A predefined data collection framework was systematically applied to extract relevant information from each included study, encompassing experimental model or participant characteristics; intervention type and dosage; treatment duration; concomitant psychological support components (preparation, dosing supervision, and integration sessions); behavioral and biological outcome measures; and mechanistic evidence of psilocin’s antidepressant effects at the molecular, cellular, neural circuit (Table 1 and Table 2), and clinical efficacy (Table 3) levels. Given the substantial heterogeneity across the included studies with respect to experimental design, animal models, clinical population characteristics, dosing regimens, and outcome measures, this review primarily employed qualitative description, with categorization, integration, and comparative analysis of similar studies to systematically delineate the multilevel evidence chain of psilocin’s antidepressant effects. Specifically, a quantitative meta-analysis of the clinical RCT data was deemed methodologically inappropriate owing to extreme clinical heterogeneity across populations (TRD, MDD, bipolar II depression, and depression with medical comorbidities), intervention protocols (single-dose vs. two-dose vs. flexible multi-dose regimens), comparators (escitalopram, niacin, low-dose psilocybin, and waitlist), and outcome metrics (MADRS, QIDS-SR-16, BDI, HAM-D) assessed at disparate timepoints (3 weeks to 24 months). This heterogeneity was further compounded by the inclusion of multiple dependent secondary reports derived from the same parent trials, thereby violating the independence assumption required for valid effect-size pooling. Consequently, clinical efficacy data were synthesized through narrative synthesis.

### 2.6. Risk-of-Bias Assessment

**Figure 2:**
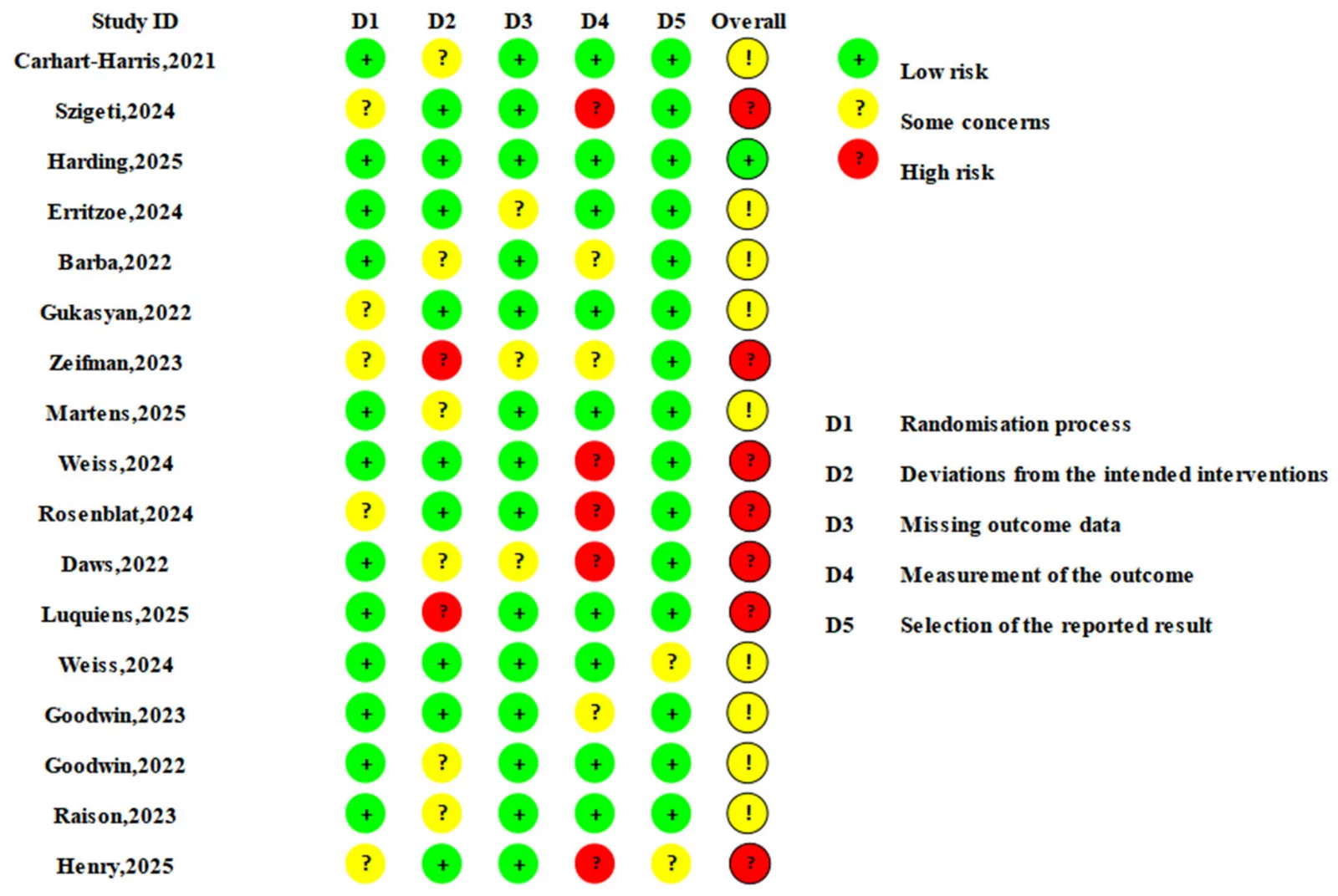
Traffic-light plot of study-level risk-of-bias assessments for the 17 included randomized controlled trials using the Cochrane RoB 2 tool. References are from [39,40,43,44,48,50,51,52,53,54,55,56,57,58,60,62,66].

**Figure 3:**
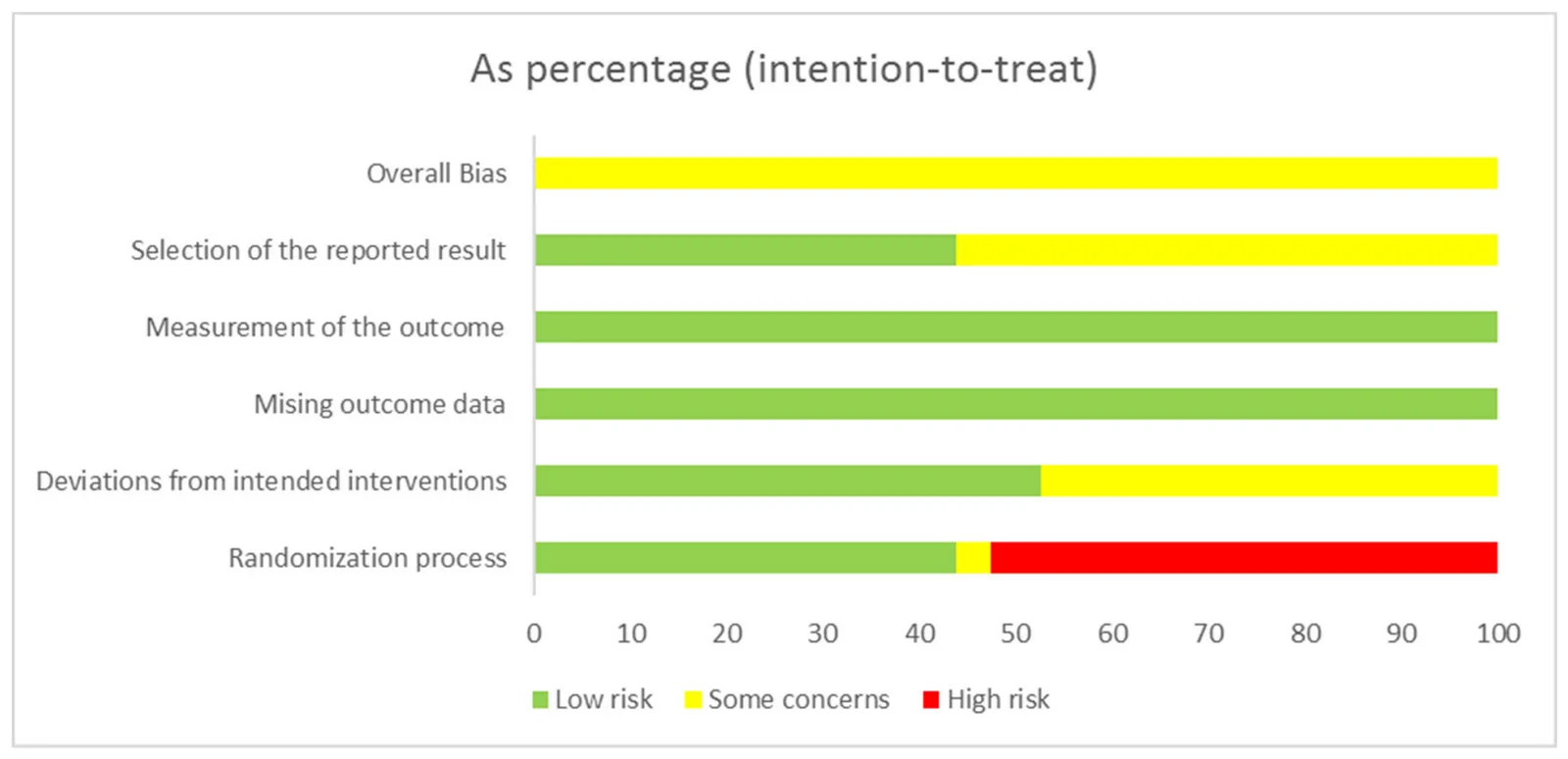
Summary of risk-of-bias assessments for the 17 included randomized controlled trials using the Cochrane RoB 2 tool.

**Figure 4:**
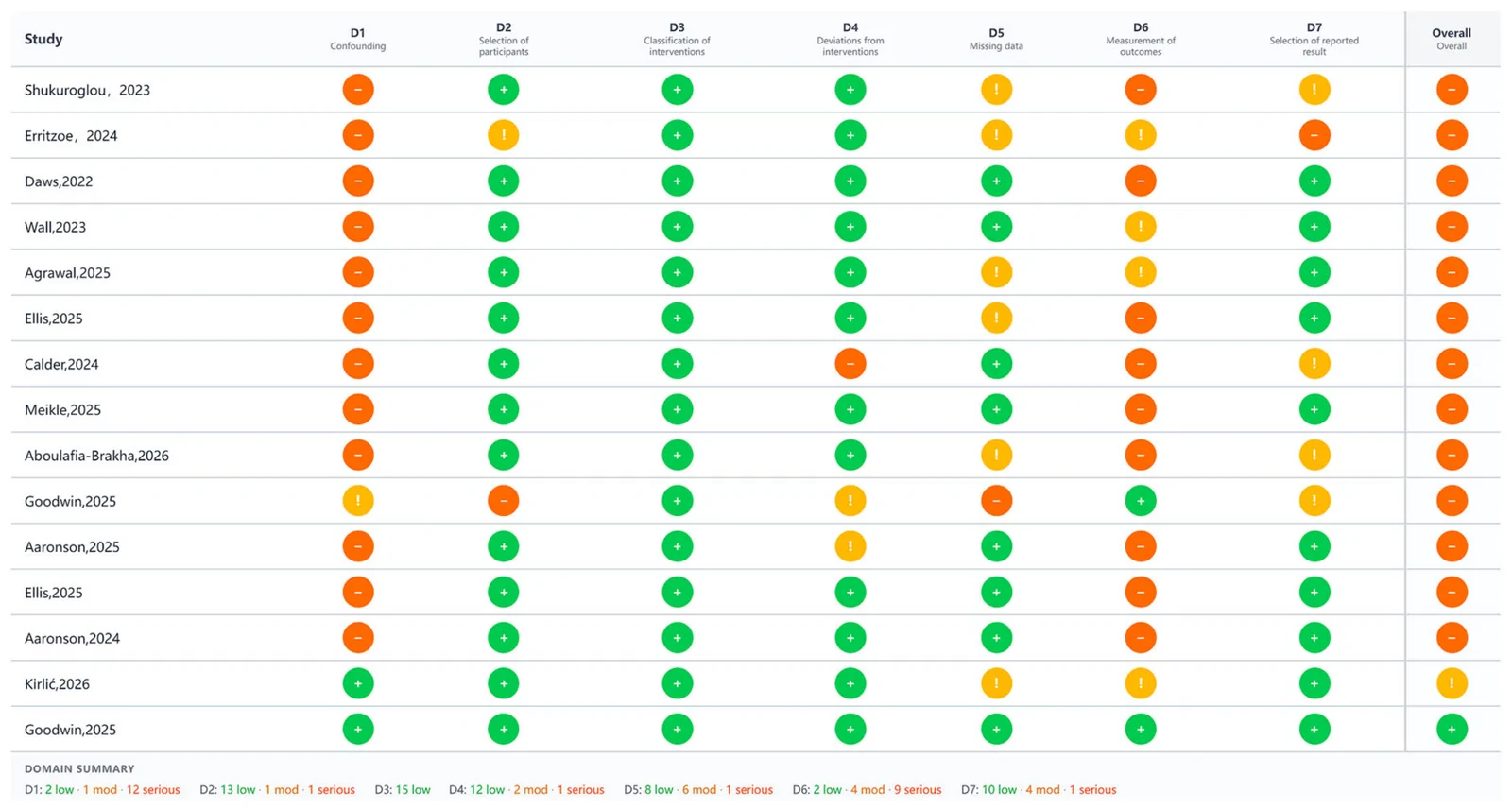
Traffic-light plot of domain-level risk-of-bias assessments for the 15 included non-randomized studies using the ROBINS-I tool. References are from [37,38,40,41,42,45,46,47,49,59,61,63,64,65,67].

**Figure 5:**
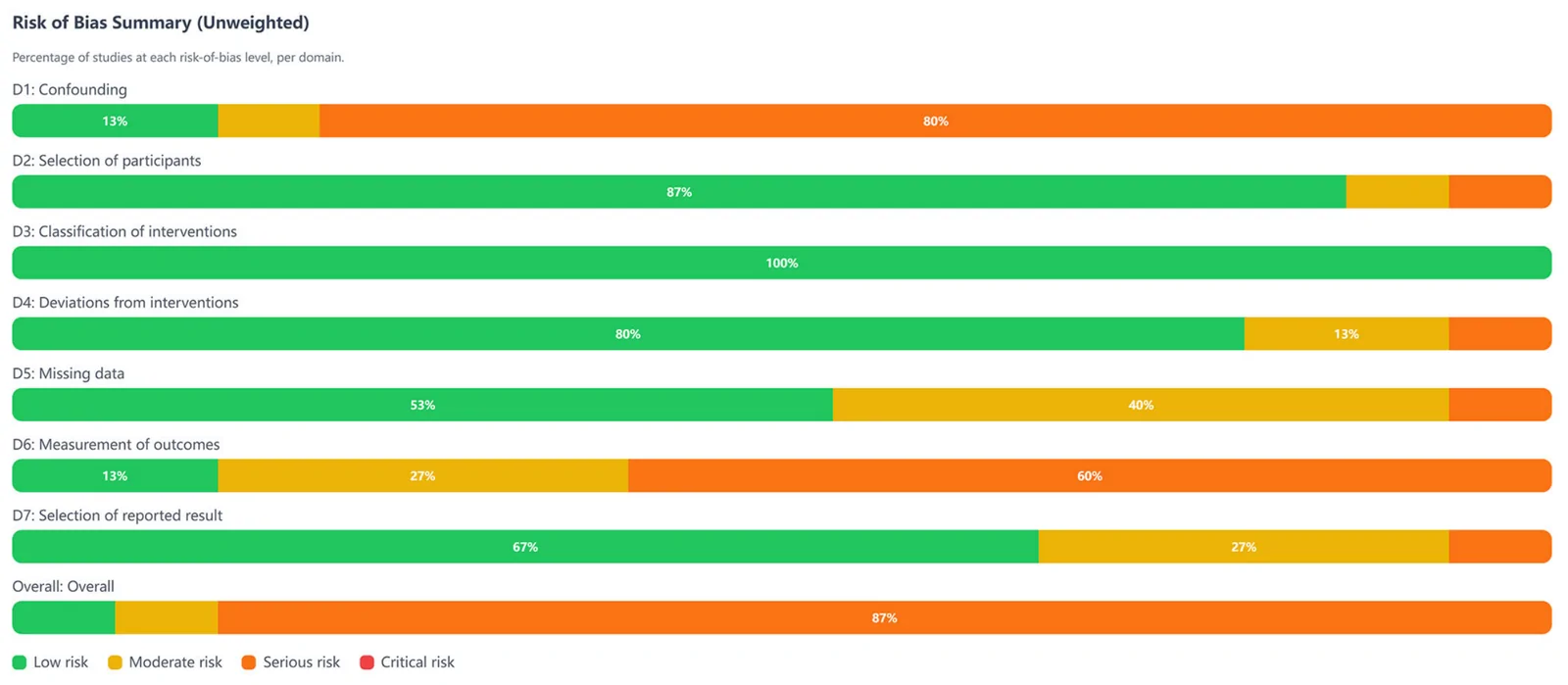
Summary of risk-of-bias assessments across seven domains for the 15 included non-randomized studies using the ROBINS-I tool.

Risk of bias was appraised using design-specific tools. Fifteen animal experimental studies were evaluated with the SYRCLE risk-of-bias tool (https://www.syrcle.network/), seventeen randomized controlled trials (RCTs) with the Cochrane Risk-of-Bias 2 (RoB 2) tool, and fifteen non-randomized studies of interventions with the ROBINS-I Version 2 (V2) tool. One included article reported an RCT and a non-randomized study; these two study designs were assessed separately with RoB 2 and ROBINS-I V2, respectively. Two independent reviewers performed all assessments, and any disagreements were resolved through discussion until consensus was reached. The SYRCLE tool assessed ten domains (Q1–Q10) for each animal study, with each domain rated as low risk (L), high risk (H), or unclear risk (U); detailed results are presented in Table 4 and Table 5. The RoB 2 tool evaluated five domains (D1–D5) for each RCT, with ratings of low risk (+), some concerns (?), or high risk (−); the results are summarized in Figure 2 and Figure 3. The ROBINS-I V2 tool assessed seven domains (D1–D7) for each non-randomized study, with ratings of low risk (+), moderate risk (!), or serious risk (−); the results are shown in Figure 4 and Figure 5.

## 3. Results and Discussion

### 3.1. Molecular Level

#### 3.1.1. Serotonin Receptor Target Activation

**Figure 6:**
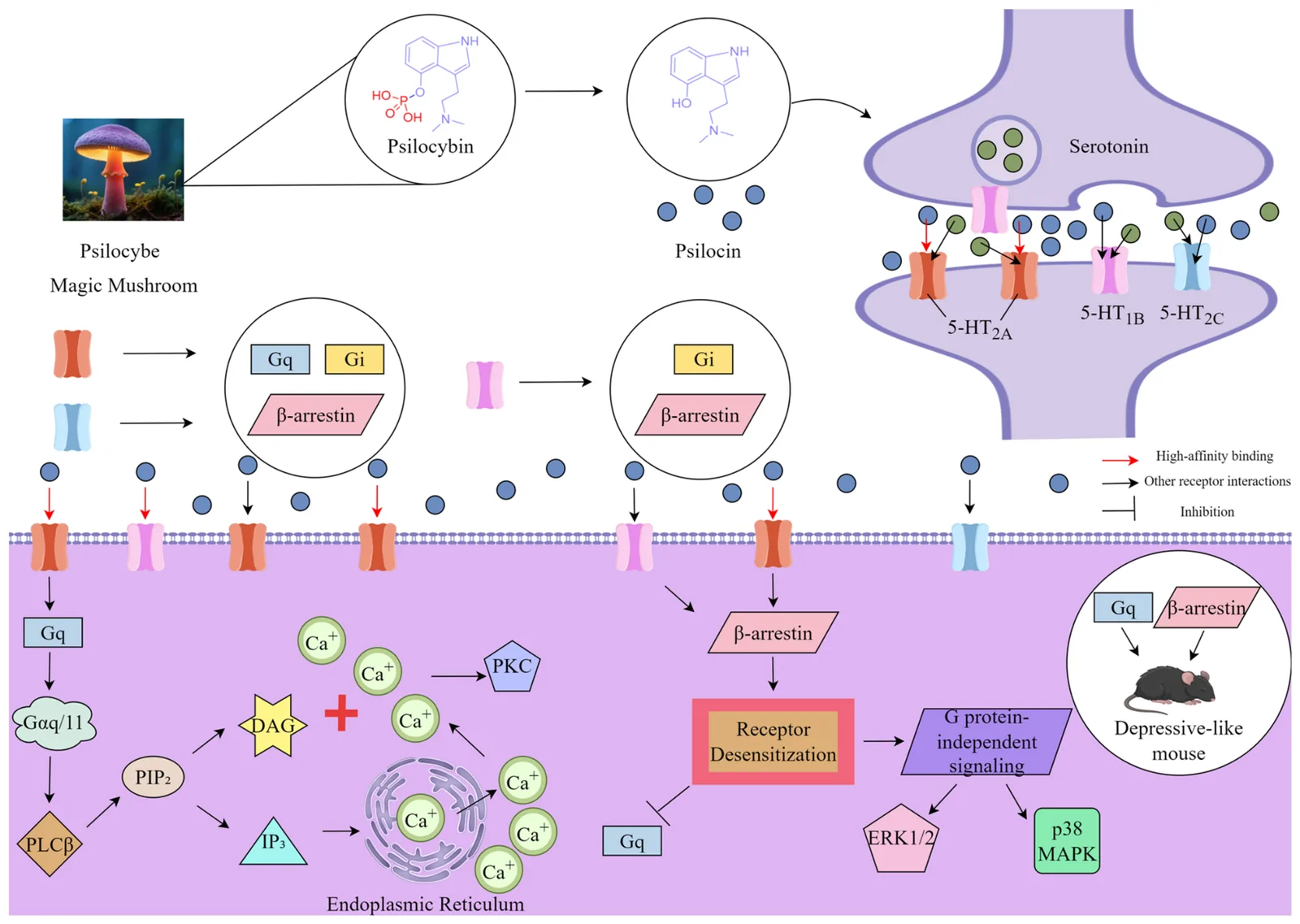
Multi-receptor 5-HT activation and intracellular signaling cascades in psilocin’s antidepressant action. PSI, the prodrug extracted from Psilocybe mushrooms, is rapidly dephosphorylated in vivo to its active metabolite psilocin. Structurally analogous to serotonin, psilocin binds to multiple serotonin receptor subtypes, with the highest affinity for 5-HT_2a_ receptors. At the 5-HT_2a_ receptor, psilocin acts as a biased agonist, engaging three canonical signaling pathways: (i) Gq/11-mediated signaling, which activates PLCβ, hydrolyzes PIP_2_ into inositol IP_3_ and DAG, triggers intracellular Ca^2+^ release from the endoplasmic reticulum, and activates PKC, ultimately promoting synaptic plasticity and potentially contributing to antidepressant effects; (ii) β-arrestin2 recruitment, which induces receptor desensitization and internalization and subsequently activates G-protein-independent signaling through the ERK1/2 and p38 MAPK pathways, contributing to sustained neuroplasticity and antidepressant action; and (iii) Gi/o signaling, which is primarily associated with the hallucinogenic effects of psilocin, with no current evidence supporting its involvement in antidepressant mechanisms. In contrast, the 5-HT_1β_ receptor—another target of psilocin—couples to Gi but lacks documented Gq engagement. Notably, 5-HT_1β_ receptors are localized presynaptically (as autoreceptors) and postsynaptically. Psilocin-mediated activation of 5-HT_2a_ receptors via the Gq and β-arrestin pathways, rather than Gi signaling, constitutes the molecular basis for its rapid and sustained antidepressant efficacy.

Serotonin receptors constitute a class of transmembrane proteins; the majority belong to the G-protein-coupled receptor (GPCR) superfamily, whereas the 5-HT_3_ receptor represents a ligand-gated ion channel [68]. GPCR-type serotonin receptors specifically recognize and respond to the endogenous neurotransmitter 5-HT signals, activating downstream G-protein subtypes and β-arrestin-mediated signal transduction pathways (Figure 6) to broadly participate in mood regulation, stress adaptation, and cognitive flexibility; dysfunction of these receptors underlies the pathology of depression [69]. Although structurally analogous to 5-HT, psilocin exhibits distinct receptor-binding patterns and signal transduction characteristics. Current preclinical evidence indicates that psilocin’s antidepressant effects through the serotonin receptor system demonstrate receptor subtype selectivity and effect dependency, with primary molecular targets involving 5-HT_2a_ and 5-HT_1β_ receptor subtypes.

Among 5-HT receptor subtypes, 5-HT_2a_ receptors demonstrate the highest concentrations in the cortical, hippocampal, and amygdalar regions [70], and extensive research indicates that 5-HT_2a_-receptor-mediated Gq signaling pathways play a pivotal role in psychedelic antidepressant-like effects [71]. However, the biased agonism of psychedelics at 5-HT_2_AR requires further examination. Three animal experimental studies support psilocybin-derived antidepressant effects through specific 5-HT_2a_ receptor activation, including three rat paradigm studies [22,23,25], all assessing acute effects of PSI. To investigate cognitive flexibility, Torrado Pacheco et al. [22] administered acute intraperitoneal PSI (1 mg/kg) to 49 Long–Evans rats; of these, 35 underwent set-shifting tasks involving co-administration of either the 5-HT_2a_ antagonist ketanserin or the 5-HT_2_c antagonist SB242084, whereas 13 underwent reversal learning tasks comparing PSI to saline. The results demonstrated that psilocin, derived from administered PSI, significantly enhanced set-shifting ability through 5-HT_2a_ receptor activation, suggesting that 5-HT_2a_-receptor-mediated cognitive flexibility improvement may constitute an antidepressant adjunctive mechanism [22]. In receptor expression regulation, Erkizia-Santamaría et al. examined two intraperitoneal PSI injections (1 mg/kg each, 7-day interval) in 64 male C57BL/6J chronic unpredictable mild stress (CUMS) mice, revealing that psilocin reversed CUMS-induced anhedonia and behavioral despair while significantly upregulating cortical 5-HT_2a_ receptor expression, implicating receptor upregulation in its antidepressant mechanism [23]. Regarding dose–effect relationships, Wojtas et al. [25] investigated subcutaneous PSI administration at 2 and 10 mg/kg in adult male Wistar-Han rats. The study employed brain microdialysis to assess limbic neurotransmitters, Western blotting to evaluate receptor density, and open field tests to measure anxiety-like behaviors. Psilocin bidirectionally regulated hippocampal 5-HT_2a_ receptor protein levels measured 7 days post-administration—decreasing expression at 2 mg/kg and increasing it at 10 mg/kg—while both doses acutely elevated nucleus accumbens dopamine and 5-HT release. In the open field test, psilocin increased time spent in the central zone and decreased peripheral exploration (thigmotaxis), reflecting anxiolytic-like effects; notably, this central preference persisted at 24 h when locomotor suppression had resolved. Although the open field test primarily indexes anxiety-like responses, the observed NAc monoamine release and limbic GABAergic modulation may provide a neurobiological substrate relevant to psilocin’s broader therapeutic effects on stress, anxiety, and mood disorders [25]. However, not all of psilocin’s antidepressant effects strictly depend on 5-HT_2a_ receptor activation [24]. Hesselgrave et al. [24] investigated single-dose intraperitoneal PSI (1 mg/kg) in male C57BL/6J CUMS mice, employing sucrose preference tests, female urine preference tests, and hippocampal CA1 field potential recordings. Psilocin reversed CUMS-induced anhedonia and enhanced hippocampal TA-CA1 synaptic AMPA/NMDA ratios—effects not blocked by the 5-HT_2a_/_2_c antagonist ketanserin—suggesting 5-HT_2a_-receptor-independent antidepressant mechanisms [24].

Furthermore, within the GPCR family, 5-HT_1β_ receptors are widely distributed in the basal ganglia, where they function primarily as presynaptic autoreceptors inhibiting serotonin release, while also exhibiting postsynaptic localization in certain circuits [72,73]. Both configurations represent targets implicated in the mechanisms of conventional antidepressant medications. Fleury et al. investigated 5 mg/kg PSI in 5-HT_1β_-receptor-knockout mice (originally generated on a C57BL/6 background [74]) and littermate controls, with 155 mice subjected to chronic corticosterone stress paradigms followed by gustometer sucrose-licking assessment of anhedonia. Psilocin significantly reversed stress-induced sucrose-licking reduction—an effect abolished in 5-HT_1β_-receptor-knockout mice—establishing 5-HT_1β_ receptors as essential molecular substrates for psilocin’s reversal of anhedonia, a core depressive symptom [26].

In summary, five preclinical studies elucidate the molecular targeting profile of psilocin’s antidepressant action across different receptor subtypes: 5-HT_2a_ receptors constitute the primary target, with three independent studies providing convergent support across multiple experimental paradigms [22,23,25]; 5-HT_1β_ receptors represent essential auxiliary targets, with gene-knockout experiments providing causal evidence [26], while non-absolute dependence on 5-HT_2a_ receptors suggests potential multi-receptor synergy or downstream signaling pathway compensatory activation mechanisms [24].

#### 3.1.2. Critical Regulation of Serotonin Transporters

The serotonin transporter (5-HTT), responsible for serotonin reuptake, functions as the “valve” maintaining 5-HT signaling homeostasis. Excessive transporter activity or abnormal expression leads to excessive synaptic 5-HT clearance and diminished signal transduction—alterations that have been associated with depressive-like behaviors in animal models [75]. Gattuso et al. [27] investigated the effects of a single intraperitoneal dose of PSI (1 mg/kg) in 5-HTT-knockout C57BL/6J mice and wild-type controls, using the forced swim test. In this study, only total immobility time during the last 4 min was quantified via automated scoring, without differentiation between swimming, climbing, or other active coping behaviors. PSI failed to significantly alter the immobility duration across genotypes, indicating the absence of antidepressant-like effects in these animals [27].

This study suggests that 5-HTT functional integrity may be necessary for psilocin’s acute behavioral effects, as psilocin failed to produce typical antidepressant-like responses in mice lacking the transporter entirely [27]. This lack of response likely reflects lifelong compensatory adaptations in serotonergic tone—such as 5-HT receptor downregulation, desensitization, or downstream signaling remodeling—driven by chronic extracellular 5-HT elevation in constitutive knockout animals. These findings do not necessarily indicate that 5-HTT is an obligate molecular target or that its acute inhibition would similarly abolish psilocin’s efficacy. Furthermore, because constitutive knockout eliminates the gene completely, this model represents an extreme scenario that may overstate the transporter’s absolute requirement; a partial knockdown model would more closely mirror patient pathophysiology, where 5-HTT is transcriptionally or functionally suppressed. Finally, exclusive reliance on a global immobility score represents a relatively crude metric that may obscure more nuanced drug responses [76].

#### 3.1.3. Molecular Remodeling of Synaptic Plasticity-Related Signaling Pathways

Depression-related synaptic plasticity deficits are closely associated with BDNF-TrkB and mTOR signaling pathway dysregulation [77,78]. Psilocin activates TrkB receptors and BDNF signaling pathways, rapidly promoting dendritic spine remodeling and synaptic protein synthesis. Four animal experimental studies evaluated psilocin’s effects on depressive-like behaviors and synaptic plasticity via the upregulation of BDNF and its high-affinity receptor, TrkB [28,29,30,31], with two studies from the same team additionally examining ERK, Akt, and mTOR signaling pathways [28,29] and one further examining synaptic protein levels and dendritic spine remodeling in a chronic corticosterone model [31]. Wang et al. [28] investigated 1 mg/kg PSI in 16 male Wistar rats subjected to chronic social instability stress (SIS) combined with predator odor exposure (POE) to model stress-induced depressive-like behaviors, assessing behavioral despair and cognitive function. Early administration significantly restored SIS-induced serum BDNF decline and upregulated BDNF expression across the prefrontal cortex, amygdala, hypothalamus, and other brain regions, while elevating TrkB levels in the PFC, AMG, HIP, and HYP, suggesting BDNF-TrkB-pathway-mediated antidepressant-like effects. This study further demonstrated psilocin-enhanced ERK phosphorylation in the prefrontal cortex, hippocampus, and hypothalamus; Akt phosphorylation in the amygdala; and mTOR phosphorylation in the prefrontal cortex and hypothalamus, demonstrating differential ERK, Akt, and mTOR signaling pathway activation across brain regions [28]. Additionally, this team evaluated single-dose intraperitoneal PSI (1 mg/kg) in 22 male Wistar-Kyoto (WKY) rats, modeling treatment-resistant depressive-like features and assessing the expression of proteins involved in the BDNF, TrkB, and downstream ERK/Akt/mTOR signaling pathways in the prefrontal cortex, amygdala, hippocampus, and hypothalamus via Western blot and ELISA. Early PSI intervention significantly upregulated TrkB expression in the hippocampus and amygdala; increased BDNF levels in the prefrontal cortex, hippocampus, and hypothalamus; and differentially activated ERK, Akt, and mTOR phosphorylation across brain regions, indicating BDNF-TrkB signaling pathway promotion and downstream molecular remodeling in psilocin’s antidepressant action [29]. Kolasa et al. [30] investigated psilocin’s effects on synaptic plasticity in TRD, administering single-dose intraperitoneal PSI (0.3 mg/kg) to male WKY and WIS rats and detecting prefrontal cortical BDNF mRNA, neurotrophic factor receptor tyrosine kinase 2 (Ntrk2) mRNA encoding TrkB receptors, and serum BDNF protein levels. Psilocin significantly affected BDNF mRNA expression levels and specifically upregulated neurotrophin-4 (Ntf4) mRNA expression in TRD-like paradigm WKY rats, without altering serum BDNF levels; significant strain differences existed in Ntrk2 gene expression between WKY and WIS rats, although psilocin failed to significantly alter Ntrk2 expression at the mRNA level. These findings suggest that psilocin regulates BDNF-, Ntf4-, and TrkB-related signaling pathways to modulate synaptic plasticity in TRD [30]. Zhao et al. administered single-dose psilocybin (2.5 mg/kg, i.p.) to chronic CORT-exposed male C57BL/6J mice (n = 6/group for molecular analyses) and found that it rescued BDNF-TrkB-mTOR signaling and restored synaptic protein levels (p-GluA1, PSD95, synapsin-1) in the PFC and hippocampus [31].

In summary, these four studies collectively reveal an endotype-dependent, dual-hierarchy plasticity cascade underlying psilocin’s antidepressant action. In the exogenous stress-sensitive model [28,31], early psilocin intervention suppresses HPA-axis hyperactivity and drives systemic protein-level restoration of BDNF–TrkB alongside broad ERK/Akt/mTOR phosphorylation [28], while also restoring synaptic protein expression and dendritic structural integrity in the PFC and hippocampus [31]. Conversely, in the endogenous TRD-like paradigm using WKY rats [29,30], psilocin rescues TSH and elicits transcriptional reprogramming (Ntf4/Arc upregulation), with divergent effects on serum BDNF. This dissociation suggests that psilocin deploys acute protein restoration and phosphorylation signaling in stress-reactive states vs. delayed transcriptional remodeling in endogenous TRD, providing a mechanistic rationale for its single-dose sustained efficacy and for patient stratification.

### 3.2. Cellular Level

Neurons, as the cellular substrate of emotional and cognitive brain functions, receive, integrate, and transmit neural signals. Depression’s pathogenesis involves structural and functional abnormalities across multiple brain regions, with neuronal populations including prefrontal cortex (mPFC) glutamatergic neurons, central amygdala (CeA) neurons, and orbitofrontal cortex (OFC) neurons receiving particular attention. Previous studies demonstrate that mPFC astrocyte–neuron glutamine–glutamate cycling imbalance, selective CeA glutamatergic neuron SIRT1 knockdown, and OFC neuronal hyperactivity are closely associated with the generation of depression-like behaviors [79,80,81]. Based on this foundation, we summarize psilocin’s antidepressant effects through action on specific neuronal cell populations.

Two studies evaluated psilocin’s antidepressant mechanisms through induction of neural cell plasticity responses [32,33], both employing Sprague Dawley (SD) rats. Funk et al. [32] investigated the effects of single-dose subcutaneous PSI (0.1–3 mg/kg) on neuronal cell activation in the mPFC, CeA, and other brain regions in 32 male SD rats. Psilocin dose-dependently increased neuronal cell activation across these regions—most prominently in the CeA, where approximately 50% of neurons were activated by high-dose PSI vs. approximately 10% in controls. These findings highlight the CeA as a prominent site of psilocin-induced neuronal activation, although this does not necessarily establish the CeA as a causal initiating node for antidepressant efficacy, given its well-established roles in fear, threat detection, and general emotional arousal [32]. García-Cabrerizo et al. [33] evaluated the effects of repeated oral PSI (0.3 and 1 mg/kg) on hippocampal neurogenesis and behavior in 60 adolescent SD rats. The 1 mg/kg dose significantly increased hippocampal NeuroD1^+^ neural progenitor cell numbers, with antidepressant-like behavioral improvement showing a significant positive correlation with NeuroD1^+^ cell counts, suggesting that psilocin may exert antidepressant-like effects through the promotion of hippocampal neurogenesis as a cellular plasticity mechanism [33]. One study employed causal validation through neuronal target knockdown, identifying specific cell types as essential for psilocin’s antidepressant-like effects [34]. Huang et al. [34] investigated the effects of single-dose intraperitoneal PSI (1 mg/kg) on OFC neurons in 33 male C57BL/6J mice. The study assessed depressive-like behaviors through repeated forced swim tests and utilized AAV-mediated Cre-dependent shRNA to specifically knock down 5-HT_2a_ receptor mRNA (Htr2a) in OFC layer-5 pyramidal neurons or PV-positive interneurons. Knockdown of Htr2a in pyramidal neurons abolished psilocin’s antidepressant-like behavioral effects, while knockdown in PV neurons only partially attenuated these effects, establishing OFC layer-5 pyramidal neurons and Htr2a as essential cellular targets for psilocin’s behavioral responses [34].

In summary, these three studies collectively delineate a hierarchical landscape of psilocin’s cellular actions rather than merely listing parallel findings [32,33,34]. Funk et al. [32] revealed dose-dependent neuronal activation across the CeA and mPFC, yet such widespread activation likely reflects acute emotional and arousal-related engagement—consistent with the CeA’s established roles in fear and threat detection—rather than a depression-specific causal trigger. García-Cabrerizo et al. [33] demonstrated that repeated psilocybin administration promotes hippocampal neurogenesis (Ki-67, NeuroD1, BrdU), providing a structural plasticity substrate for sustained behavioral improvements. Most critically, Huang et al. [34] established through targeted Htr2a knockdown that OFC layer-5 pyramidal neurons—but not PV+ interneurons—are essential for the observed behavioral effects, moving from correlation to causality. Collectively, these findings support a functional tripartite framework: widespread activation reflects acute pharmacological engagement, hippocampal neurogenesis supports long-term resilience, and circuit-specific necessity in the OFC pinpoints critical effector nodes. This differentiation between correlative activation, structural remodeling, and causal dependency underscores that psilocin’s actions are brain-region- and cell-type-selective, offering a roadmap for precision targeting.

### 3.3. Neural Circuit Level

Neural circuits regulating emotion, reward, and executive function depend on coordinated connectivity and information integration across brain regions; circuit dysfunction may induce depression [82], while restoring normal integration states is central to antidepressant intervention.

**Figure 7:**
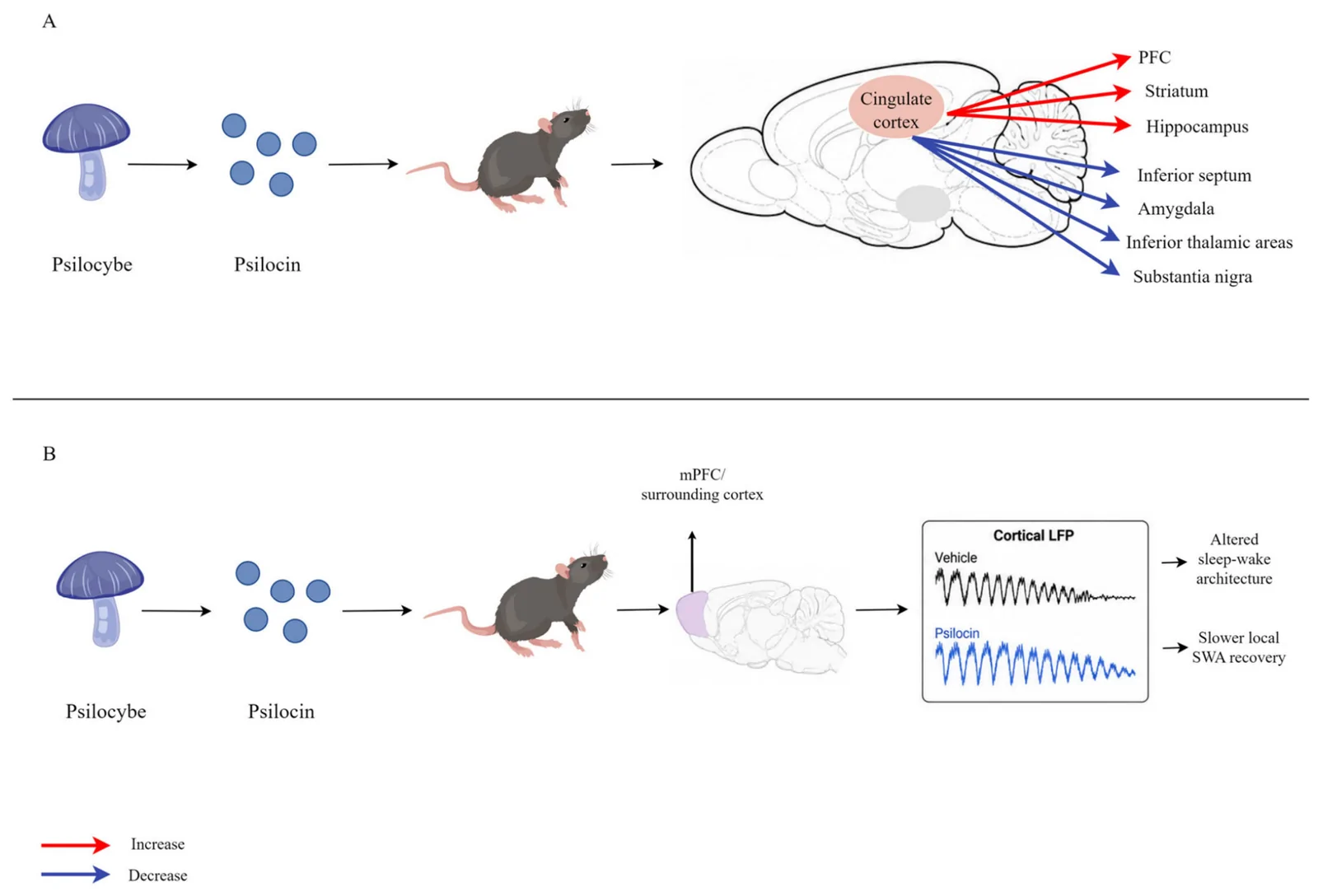
Rodent neural circuit and cortical electrophysiological alterations associated with psilocin exposure: (**A**) In rats, acute psilocin hydrochloride induces cingulate-cortex-centered functional connectivity remodeling, with increased connectivity between the cingulate cortex and the prefrontal cortex (PFC), striatum, and hippocampus (red arrows) and decreased connectivity with the inferior septum, amygdala, inferior thalamic areas, and substantia nigra (blue arrows). (**B**) In mice, acute psilocin alters sleep–wake architecture and slows the local recovery of sleeping slow-wave activity (SWA) in LFPs recorded from the medial prefrontal and surrounding cortex, without corresponding changes at the global EEG level. Both panels illustrate rodent-specific findings; their direct contribution to antidepressant-like behavior remains to be established.

Two preclinical rodent studies evaluated psilocin’s effects on neural circuit activity and functional connectivity [35,36]. In an exploratory, small-scale electrophysiology study (n = 8), Thomas et al. found that acute psilocin delayed REM sleep onset, impaired NREM sleep maintenance for up to approximately 3 h, and enhanced a 3–5 Hz oscillation in the frontal EEG and in local field potentials (LFPs) recorded from the medial prefrontal and surrounding cortex [35]. When combined with 4 h of sleep deprivation, psilocin did not alter global EEG slow-wave activity rebound, but it significantly slowed the recovery rate of sleeping slow-wave activity in prefrontal LFPs, providing preliminary evidence that psilocin locally alters sleep–wake homeostasis in the prefrontal cortex [35] (Figure 7B). Liu et al. [36] investigated the effects of a single intraperitoneal dose of PSI (2 mg/kg) on functional connectivity between cortical and subcortical regions in 91 male SD rats, using resting-state BOLD fMRI to calculate whole-brain degree centrality, ROI-wise functional connectivity, and cingulate seed-based functional connectivity, with complementary EGR1 immunofluorescence. Using the anatomical cingulate cortex as the seed, psilocin increased cingulate connectivity with the orbital/prefrontal cortex, striatum, and hippocampus, while decreasing connectivity with the inferior part of the septum, amygdala, inferior thalamic areas, and substantia nigra. These network-level changes were accompanied by widespread EGR1 upregulation across cortical, striatal, and hippocampal regions, suggesting that psilocin remodels cingulate-cortex-centered corticostriatal–hippocampal functional connectivity [36] (Figure 7A).

Four clinical trials evaluated psilocin’s antidepressant effects through brain network functional remodeling [37,38,39,40]. Shukuroglou et al. [37] identified significantly enhanced music-evoked pleasure in 19 patients with TRD following PSI treatment, which was negatively correlated with improvements in anhedonia scores (SHAPS). Functional connectivity analysis revealed significantly attenuated nucleus accumbens (NAc) coupling with DMN-like regions post-treatment (Figure 7B), suggesting that psilocin restores reward-system function through the release of hyperinhibition between the DMN and the ventral striatum [37]. Wall et al. employed low-frequency amplitude (ALFF) analysis in 19 patients with treatment-resistant depression to demonstrate significantly enhanced bilateral superior temporal gyrus (STG) neural activity while listening to music following PSI treatment, positively correlated with acute subjective drug-effect intensity, indicating enhanced auditory cortex emotional processing capacity [38]. Daws et al. [40] conducted an open-label trial in 16 patients with TRD with escalating oral PSI (10 mg and 25 mg), performing fMRI one day after the second administration. The results demonstrated significantly reduced whole-brain network modularity, indicating elevated global functional integration following PSI treatment, where the magnitude of modularity reduction was significantly correlated with 6-month BDI score improvement. Network cartography further revealed attenuated DMN internal connectivity alongside enhanced cross-network integration between the DMN and the executive network (EN) and salience network (SN). This suggests that psilocin may correct depression-related functional network segregation by reducing brain network modularization, restoring cognitive flexibility to exert antidepressant effects [40]. Harding et al. [39] compared PSI with escitalopram using a musical surprise task, finding significantly superior anhedonia improvement in the PSI group, with enhanced music-evoked “vitality” sensations, while escitalopram reduced this positive effect. Furthermore, psilocin reduced ventromedial prefrontal cortex (vmPFC) responsiveness to musical surprises while enhancing visual and auditory sensory cortex activation and attenuating angular gyrus activation, suggesting that psilocin shifts processing from excessive interoceptive to exteroceptive drive through reduced higher-order cortical salience weighting of prediction errors; conversely, escitalopram enhanced the activation of memory and emotion-processing regions, presenting “emotional blunting” characteristics [39].

Beyond DMN-centric changes, evidence from broader psychedelic research suggests that psilocybin may also engage network reorganization encompassing enhanced DMN–ECN and DMN–SN cross-network integration, disrupted corticostriatal and thalamocortical gating, increased brain entropy, and remodeling of socio-emotional circuits [83,84].

In summary, two preclinical animal studies reveal psilocin’s capacity to modulate prefrontal cortical local rhythmic homeostasis and enhance cingulate–striatal–hippocampal large-scale functional connectivity [35,36]. Four clinical trials further validate these findings at the human brain network level: post-PSI-treatment DMN–ventral striatum hyperinhibition release, enhanced auditory cortex emotional processing, reduced whole-brain network modularity with increased executive network dynamic flexibility, and processing mode shifts from excessive interoception to exteroceptive drive [37,38,39,40]. These six studies provide complementary, scale-spanning observations—from local rhythmic homeostasis in rodents to whole-brain network reorganization in humans—suggesting that psilocin may exert antidepressant effects through multilevel neural circuit remodeling, although direct cross-species homology cannot be assumed [35,36,37,38,39,40].

### 3.4. Clinical Efficacy

#### 3.4.1. Therapeutic Effects of PSI-Assisted Treatment in Depression

While multiple preclinical studies have preliminarily revealed psilocin’s rapid and sustained antidepressant potential, the primary translational question concerns whether PSI-assisted treatment achieves antidepressant efficacy in patients with depression. We systematically summarize PSI’s therapeutic effects as administered in clinical trials—that is, within a framework of structured psychological support. This analysis offers dual advantages: maximally preserving PSI’s original pharmacological characteristics to authentically reflect its direct effects on core depressive symptoms, cognitive function, and emotional regulation following PSI administration within this supported framework; and clarifying dose–effect relationships to provide reliable foundations for subsequent precise dose optimization and individualized treatment. However, it is important to note that all clinical trials included in this review administered PSI in conjunction with structured psychological support, typically encompassing preparatory sessions, professional monitoring during dosing, and post-administration integration sessions; therefore, the efficacy summarized below reflects psilocybin-assisted treatment rather than the pharmacological effect of PSI in isolation.

Thirteen studies evaluated the antidepressant effects of PSI-assisted treatment across depressive disorders, encompassing unipolar depression (MDD and TRD) and bipolar II depression, across various timepoints from the acute phase to long-term follow-up, confirming that single- or limited-dose administration rapidly and persistently alleviates depressive symptoms [41,42,43,44,45,46,47,57,59,60,61,62,63,67].

An open-label pilot trial (ClinicalTrials.gov: NCT04433858) including 15 U.S. veterans with severe TRD reported short-term [41] and long-term [42] outcomes following single-dose 25 mg PSI. The initial publication focused on short-term efficacy from 3 weeks to 12 weeks, demonstrating 60% response and 53% remission at 3 weeks, with partial maintenance at 12 weeks [41]. A subsequent follow-up analysis of the same cohort (10 of the original 15 participants) extended observation to 12 months, showing that while antidepressant effects began to wane after 6 months, significant symptom alleviation persisted across 12 months [42].

Two studies evaluated the effects of PSI in patients with depression and comorbid conditions [59,60]. Agrawal et al. [59] assessed single-dose 25 mg PSI combined with psychological support for long-term efficacy in 30 patients with MDD and cancer, employing the Montgomery–Åsberg Depression Rating Scale (MADRS) and the Hospital Anxiety and Depression Scale (HADS) as primary assessment instruments. Fifty percent of patients maintained depression remission at 24 months, with 42.9% demonstrating sustained anxiety symptom reduction [59]. Luquiens et al. [60] evaluated two 25 mg PSI sessions assisted by psychotherapy for short-term efficacy in 30 patients with depression and comorbid alcohol-use disorder (AUD), employing the BDI, Timeline Followback (TLFB), and Craving Experience Questionnaire (CEQ) as primary assessment instruments. The 25 mg group demonstrated significantly higher abstinence rates at 12 weeks vs. controls, with significant reductions in depressive symptoms and alcohol cravings [60].

Goodwin et al. reported the phase 2b randomized controlled trial COMP001 (NCT03775200), which evaluated single-dose 25, 10, and 1 mg PSI in 233 patients with TRD, with the primary report demonstrating significant superiority of 25 mg over 1 mg in MADRS score reduction at 3 weeks [43]. Secondary analyses from the same trial explored patient-reported outcomes (QIDS-SR, GAD-7, SDS, WSAS) [44] and dose–experience–efficacy relationships [45]. A 52-week observational follow-up of COMP001 completers (COMP004; NCT04519957) further examined long-term durability, showing prolonged time to depressive event in the 25 mg group [47].

The four studies each emphasized distinct aspects of PSI TRD treatment [45,46,61,63]. Meikle et al. evaluated the safety and efficacy of PSI combined with psychological support for TRD [61]. Aaronson et al. focused on single high-dose 25 mg PSI application in severe TRD [63]. Kirlić et al. retrospectively analyzed relationships between patient pretreatment clinical characteristics and PSI acute experience quality [46]. Goodwin et al. reported pre-planned exploratory and post hoc analyses of COMP001 data, examining relationships between PSI dose, subjective experience, and therapeutic effect [45]. Specifically, Meikle et al. employed mixed methods to evaluate the efficacy and safety of PSI combined with psychological support; they utilized the QIDS-SR to quantify changes in depressive symptoms, the WHO Quality of Life-Bref (WHOQoL-Bref) to assess quality-of-life improvements, and the 11-Dimensional Altered States of Consciousness Scale (11D-ASC) to explore dimensions of subjective experience, thereby achieving a multidimensional assessment of symptoms, function, and experience [61]. Aaronson et al. focused on the application of a single high dose (25 mg) of PSI in severe TRD, using MADRS as the core efficacy indicator; they systematically recorded adverse events and safety data, with particular attention paid to suicidal ideation and other severe adverse events, providing critical evidence regarding the long-term safety of high-dose PSI [63]. Kirlić et al. innovatively employed the Five-Dimensional Altered States of Consciousness Scale (5D-ASC) and Emotional Breakthrough Inventory (EBI) for retrospective quantification of relationships between pretreatment clinical characteristics and PSI acute experience quality, revealing clinical characteristics’ predictive effects on subjective experiences [46]. Goodwin et al. analyzed COMP001 trial data to compare efficacy differences between 25, 10, and 1 mg doses of PSI, utilized fMRI technology to observe pre- and post-treatment changes in brain functional connectivity, and analyzed dose-dependent relationships between subjective experiences and efficacy using the 5D-ASC, thereby providing comprehensive evidence for PSI dose optimization and mechanistic research [45]. Extending the multi-dose approach to MDD, Gukasyan et al. prospectively followed 24 patients with moderate-to-severe MDD for 12 months after two psilocybin doses (20 mg/70 kg and 30 mg/70 kg, approximately 2 weeks apart) with supportive psychotherapy, demonstrating sustained large antidepressant effects (Cohen’s d = 2.4), with 75% response, 58% remission, and no serious adverse events [62].

Two studies extended PSI-assisted treatment evaluation to bipolar II (BDII) depression—a population historically excluded from psychedelic trials due to concerns regarding affective switching. Aaronson et al. [67] conducted an open-label non-randomized controlled trial with 15 participants with treatment-resistant bipolar II major depressive episodes, administering a single 25 mg dose of synthetic psilocybin with psychotherapy. At 3 weeks, the mean MADRS score decreased by 24.00 points (Cohen’s d = 4.08; *p* < 0.001), yielding an 80% remission rate; benefits persisted to 12 weeks without manic/hypomanic conversion. Rosenblat et al. [57] reported a randomized waitlist-controlled feasibility trial with 30 TRD participants (including 4 BDII patients), demonstrating significant MADRS reduction at 2 weeks (Hedges’ g = 1.07, *p* < 0.01) and no serious adverse events in the BDII subsample. These preliminary findings suggest that single- or limited-dose psilocybin rapidly alleviates depressive symptoms in BDII depression, with 25 mg demonstrating robust antidepressant effects and no signal of affective switching [57,67].

In summary, these studies explore optimal PSI antidepressant treatment doses from dose–effect association perspectives [41,42,43,44,45,46,47,57,59,60,61,62,63,67]. Reports from the COMP001 trial [43,44,45] and its separate 52-week observational follow-up (COMP004) [47] consistently confirm 25 mg PSI’s significant superiority over 10 and 1 mg in the treatment of TRD symptoms. A retrospective analysis of the same COMP001 cohort further examined pretreatment predictors of the acute psychedelic experience [46]. Although four studies employed only 25 mg of PSI, the results demonstrated significant efficacy, evidenced by marked improvements in depressive symptoms [41,42,59,63]. Three studies employing two-dose PSI regimens confirm effective improvement in depressive symptoms [60,61,62]. In the BDII subgroup, two studies provide preliminary evidence for rapid symptom alleviation following single-dose 25 mg PSI without manic/hypomanic conversion; however, both are constrained by small sample sizes and open-label or waitlist-controlled designs, limiting causal inference and requiring larger randomized controlled trials to establish efficacy in this population [57,67].

#### 3.4.2. Efficacy Comparison: PSI vs. Escitalopram

In clinical antidepressant treatment, SSRIs are widely utilized due to their moderate effects, favorable tolerability, and high safety profiles [85], but they are generally limited by onset delays (typically 2–4 weeks) and poor efficacy in patients with TRD [86,87]. In contrast, PSI achieves rapid and sustained depressive symptom relief following single-dose administration, demonstrating significant efficacy, particularly in patients with TRD. However, PSI’s clinical application as an antidepressant remains exploratory. Among SSRIs, escitalopram demonstrates relatively stable efficacy and fewer side effects, frequently serving as PSI’s comparator drug. Therefore, comparing efficacy differences between PSI and escitalopram in the treatment of MDD aims to clarify advantages and applicable boundaries of both therapeutic strategies, providing evidence-based foundations for optimizing PSI-assisted treatment protocols and achieving precise stratified treatment.

Eleven studies compared the antidepressant efficacy of PSI and escitalopram [39,40,48,50,51,52,53,54,55,56,58]. Eight studies involving patients with MDD demonstrated the superior antidepressant efficacy of PSI [39,40,50,51,52,54,55,58]; the remaining three studies found no significant efficacy differences [48,53,56].

Among the eight studies demonstrating PSI’s superiority, two employed fMRI to investigate the effects of PSI and escitalopram on brain functional networks [39,40], while four utilized QIDS-SR-16 for efficacy assessment [50,51,56,58]. Among the three studies finding no significant differences, two focused on psychological-level drug effects [53,56], while the other simultaneously assessed clinical efficacy and safety [48].

Among the eight studies demonstrating PSI’s superiority, Szigeti et al. [58] enrolled 55 patients with MDD, with the PSI group receiving two 25 mg doses 3 weeks apart combined with placebo, while the escitalopram group received daily 10 mg, escalating to 20 mg, combined with two 1 mg PSI sessions as a placebo control. After 6 weeks, the PSI group demonstrated significantly superior response rates, remission rates, and multiple mental-health secondary outcome measures compared with the escitalopram group, with confidence exceeding 95% [58]. At the neural mechanism level, Daws et al. employed fMRI in 59 patients with MDD, discovering significantly reduced brain network modularity and increased executive network dynamic flexibility following two 25 mg PSI doses 3 weeks apart, with changes significantly correlated with depressive symptom improvement, while the escitalopram group demonstrated no such alterations [40]. At the psychological experience level, Weiss et al. randomized 59 patients with MDD, finding 14 of 16 efficacy measures to be significantly superior following two 25 mg PSI doses 3 weeks apart vs. escitalopram, with core mechanisms involving PSI-induced “mystical experiences” and “ego dissolution” (absent in escitalopram treatment) [55]. At the cognitive function level, Barba et al. randomized 59 patients with MDD, confirming significant post-treatment reductions in rumination and thought suppression following two 25 mg PSI doses 3 weeks apart, while the escitalopram group demonstrated no significant thought suppression changes [51]. Henry et al. randomized 59 patients with MDD, discovering significant improvements in self-rated optimism, positive future event predictions, and dysfunctional attitudes following two 25 mg PSI doses 3 weeks apart, while the escitalopram group only exhibited reduced negative-event pessimistic expectations [54]. At the quality-of-life level, Erritzoe et al. conducted 6-month follow-up in 59 patients with MDD, finding that although both groups demonstrated comparable depression score improvements, the PSI group showed significant superiority in work and social function, psychological connectedness, and life meaning [50]. In a secondary analysis of 41 patients with MDD by Harding et al., significantly superior anhedonia score improvement was found in the PSI group (22 patients, two 25 mg doses 3 weeks apart) compared with the escitalopram group (19 patients, daily 10 mg escalating to 20 mg), accompanied by enhanced music-evoked “vitality” emotions, whereas escitalopram reduced this positive effect [39]. Zeifman et al. further randomized 59 patients with MDD, discovering PSI mental health improvement achieved through significant experiential avoidance reduction, while the escitalopram group demonstrated no significant indirect effects [52].

Conversely, three studies failed to demonstrate significant PSI–escitalopram antidepressant efficacy differences. Martens et al. randomized 59 patients with long-term MDD, finding that the PSI group (30 patients, two 25 mg doses 3 weeks apart) and the escitalopram group (29 patients, daily 10–20 mg for 6 weeks) demonstrated significantly reduced negative affective bias, with no statistically significant intergroup differences in accuracy, misclassification rates, or reaction times [53]. Weiss et al. randomized 59 patients with moderate-to-severe MDD, finding that the PSI group (30 patients, two 25 mg doses 3 weeks apart) and the escitalopram group (29 patients, daily 10–20 mg for 6 weeks) showed reductions in neuroticism, agreeableness, and impulsivity, along with increases in openness, at 6 weeks, with no statistically significant intergroup differences at any timepoint [56]. Carhart-Harris et al. randomized 59 patients with long-term MDD, finding no statistically significant differences in primary outcome (QIDS-SR-16 score changes) between the PSI group (30 patients, two 25 mg doses 3 weeks apart) and the escitalopram group (29 patients, daily 10–20 mg for 6 weeks), although some secondary outcomes trended toward PSI’s superiority without multiple comparison correction [48]. A subsequent post hoc analysis by Erritzoe et al. stratified these 59 patients by prior SSRI/SNRI discontinuation status (n = 21 discontinuers vs. n = 35 unmedicated), revealing that PSI’s superiority over escitalopram in depression and well-being outcomes was evident only in unmedicated patients; this advantage was abolished in discontinuers, who showed comparable responses across both treatments [49].

However, subsequent re-analyses of the Carhart-Harris et al. trial have challenged the interpretation of this null primary outcome. Weiss et al. identified psychometric limitations of the QIDS-SR-16—including higher variance, compound item imprecision, and lack of core depression factor focus—and demonstrated PSI’s superiority regarding depressed mood, anhedonia, and core depression through item- and factor-level reanalysis [88]. Nayak et al. further applied Bayesian methods with skeptical priors, finding strong to extremely strong evidence for PSI’s superiority on BDI-1A and HAMD-17 and extremely strong evidence for non-inferiority across all measures [89]. These findings indicate that the original null QIDS-SR-16 result reflected measurement insensitivity rather than true equivalence and that more nuanced analytical approaches consistently support PSI’s antidepressant efficacy.

In summary, although some clinical trials found no significant PSI–escitalopram differences in primary outcome measures [48,53,56], and subsequent post hoc analyses further indicated that prior SSRI/SNRI discontinuation may attenuate PSI’s relative efficacy [49], comprehensive results suggest PSI’s unique and potential advantages in the treatment of depression [39,40,50,51,52,54,55,58]. These advantages are foreshadowed by prior open-label studies in treatment-resistant depression, which demonstrated that psilocybin induces rapid and sustained symptom reduction, coupled with post-treatment amygdala hypoperfusion and reorganization of the default mode network [90,91]. PSI not only demonstrates comparable or superior depressive symptom improvement vs. escitalopram but also shows significant advantages in emotional processing enhancement, negative cognitive bias reduction, brain network reorganization, and subjective psychological experience induction. Furthermore, PSI therapy promotes patient self-insight and emotional acceptance through unique mystical experiences and emotional breakthroughs—mechanisms that may collectively contribute to PSI’s therapeutic antidepressant effects.

#### 3.4.3. Efficacy Comparison: PSI vs. Lysergic Acid Diethylamide (LSD)

Beyond escitalopram, LSD has been explored and utilized in depression treatment. LSD, an ergoline alkaloid synthesized from lysergic acid and diethylamine, shares serotonergic psychedelic properties with PSI [92]. Previous studies indicate that microdose LSD significantly improves anxiety, rumination, stress, and quality of life in patients with MDD [93]. Comparing the antidepressant efficacy of PSI and LSD helps clarify the unique mechanisms of action and clinical value of PSI among psychedelics.

Two studies evaluated the therapeutic effects of PSI and LSD in depression, finding no significant efficacy differences [64,65]. Calder et al. [64] assessed PSI and LSD’s subjective drug effects and antidepressant responses in psychedelic-assisted therapy (PAT). This study enrolled 28 patients with depression and other psychiatric disorders receiving PAT, along with 28 healthy volunteers; the patient group received total doses of 100–250 μg of LSD or 10–25 mg of PSI, while healthy controls received 100–200 μg of LSD or 15 mg of PSI. Acute drug effects were assessed using hourly real-time ratings and the retrospectively completed Mystical Experience Questionnaire (MEQ), while depressive symptom changes were evaluated using the MADRS. The results indicated no significant differences between PSI and LSD in overall drug effects and mystical experience scores, with patients demonstrating significant depressive symptom improvement and no significant efficacy differences between compounds [64]. Aboulafia-Brakha et al. [65] evaluated the efficacy and safety of PSI and LSD in patients with TRD and anxiety disorder. This study enrolled 115 patients with TRD and anxiety disorder, with 70 receiving 100 μg of LSD and 45 receiving 25 mg of PSI, employing the BDI and State-Trait Anxiety Inventory-Trait (STAI-T) for pre- and post-treatment (1–3 months) assessment of depression and anxiety symptoms. The LSD and PSI groups demonstrated significant and sustained improvements in depression and anxiety symptoms, with no significant intergroup efficacy differences [65].

In summary, both studies were conducted in Swiss medical settings, not only focusing on the potential therapeutic effects of PSI and LSD in depression treatment but also employing real-world data to evaluate both compounds’ clinical effectiveness and safety [64,65]. Calder et al. employed naturalistic data collection and analysis methods with smaller sample sizes and diverse diagnoses including depression, obsessive–compulsive disorder, bipolar disorder (BD), and social phobia [64]. Aboulafia-Brakha et al. employed retrospective analysis methods with larger sample sizes, primarily focusing on patients with MDD and anxiety disorder [65].

#### 3.4.4. Efficacy Comparison: PSI vs. Niacin

Niacin is essential for normal central nervous system function and neuronal development; consequently, it is often used as an active placebo in research on neurological and psychiatric disorders. Epidemiological evidence suggests a U-shaped association between niacin intake and depression—with moderate intake being protective, while excess intake increases risk—yet whether niacin itself exerts clinically meaningful antidepressant effects remains unproven in controlled trials [94].

Raison et al. [66] conducted a phase 2, randomized, double-blind trial across 11 US sites, involving 104 adults with MDD, comparing a single 25 mg dose of PSI with a 100 mg dose of niacin, both administered with identical psychological support. Central rater-assessed MADRS scores demonstrated significantly greater reductions in the PSI group compared with the niacin group at day 8 and at day 43. PSI also significantly improved functional disability (SDS score) and produced higher rates of sustained response (42% vs. 11%) compared with niacin [66].

In summary, this trial provides rigorous evidence that psilocybin’s antidepressant efficacy exceeds that of an active placebo control when both are delivered within an identical psychotherapeutic framework, supporting the specific therapeutic contribution of the psychedelic compound beyond contextual factors alone.

### 3.5. Risk-of-Bias Assessment of Included Studies

#### 3.5.1. Animal Experimental Studies

The methodological quality of the 15 included animal studies was appraised using the SYRCLE risk-of-bias tool (Table 4 and Table 5). Baseline characteristics (Q2), incomplete outcome data (Q8), and selective outcome reporting (Q9) were uniformly rated as low-risk, and blinding of outcome assessors (Q7) was predominantly low-risk (86.7%). However, critical limitations were evident in allocation concealment (Q3), which was unclear in 93.3% of studies; housing randomization (Q4) and outcome assessment randomization (Q6), which were unclear in all studies; and blinding of caregivers/investigators (Q5), which was unclear in 80.0% and high-risk in 13.3%. Notably, other sources of bias (Q10) were rated as high-risk in 73.3% of studies. These gaps indicate that performance and detection biases may have influenced behavioral and molecular outcomes, and the preclinical evidence should be regarded as hypothesis-generating.

#### 3.5.2. Randomized Controlled Trials

The 17 RCTs were evaluated with the Cochrane RoB 2 tool (Figure 2 and Figure 3). Missing outcome data and measurement of the outcome were uniformly rated as low-risk (100%), reflecting robust data integrity. However, the randomization process was rated as low-risk in only approximately 45% of trials, with approximately 50% classified as high-risk, and 5% raising some concerns. Deviations from intended interventions raised some concerns in 48% of trials, largely reflecting the difficulty of maintaining blinded conditions in psychedelic research. Selection of the reported result was low-risk in 43% and raised some concerns in 57%. Consequently, the overall risk of bias was rated as “some concerns” across all 17 RCTs, necessitating cautious interpretation of efficacy estimates.

#### 3.5.3. Non-Randomized Studies of Interventions

The 15 non-randomized studies were assessed with ROBINS-I V2 (Figure 4 and Figure 5). The overall risk of bias was predominantly serious (87%), with only 7% rated as low risk. Domain-level analysis identified confounding (D1) as the most critical weakness, with 80% of studies rated as serious-risk due to the absence of randomization. Measurement of outcomes (D6) also showed substantial limitations, with 60% rated as serious-risk and 27% as moderate-risk, partially attributable to the subjective nature of psychedelic experiences and open-label designs. By contrast, classification of interventions (D3) was uniformly low-risk (100%), and selection of participants (D2) was predominantly low-risk (87%). These findings indicate that non-randomized data are valuable for signal detection but limited in causal inference.

### 3.6. Discussion

#### 3.6.1. Integration of Multilevel Evidence Chains

In this review, we systematically retrieved and included 46 original studies, comprising 15 animal experimental studies—encompassing the molecular, cellular, and neural circuit levels—and 31 clinical studies, of which two were clinical neuroimaging studies on neural circuit mechanisms and 29 were clinical efficacy trials. These studies investigated the neurobiological foundations and clinical translational value of psilocin’s antidepressant effects’ from diverse dimensions. It should be noted that while the molecular, cellular, and neural circuit mechanisms discussed above are derived primarily from preclinical models, the clinical efficacy evidence was obtained within psychotherapeutic frameworks whose independent contribution, though difficult to ascertain at the neurobiological level, cannot be excluded. At the molecular level, existing evidence suggests that psilocin primarily engages 5-HT_2a_ receptor activation, 5-HT_1β_ receptor signaling, and BDNF-TrkB pathway regulation, although absolute dependence on 5-HT_2a_ receptors remains controversial. At the cellular level, psilocin may promote hippocampal neurogenesis and dose-dependently activate CeA and mPFC neurons, with OFC layer-5 pyramidal neurons serving as putative action targets. At the neural circuit level, psilocin may remodel cingulate-cortex-mediated corticostriatal–hippocampal functional connectivity and modulate prefrontal cortical sleep–wake rhythmic homeostasis. Regarding clinical efficacy, single- or limited-dose PSI administration rapidly and persistently alleviates core symptoms in patients with TRD, with 25 mg doses demonstrating significantly superior response and remission rates vs. 1 and 10 mg doses, maintaining significant efficacy at 12-month follow-up. Compared with escitalopram, PSI demonstrates comparable or superior depressive symptom improvement, with unique advantages including reductions in anhedonia, decreases in rumination, diminution of experiential avoidance, reduced brain network modularization, and induction of subjective mystical experiences. Compared with LSD, no significant differences exist in overall antidepressant efficacy. In contrast, compared with niacin, a single 25 mg dose of psilocybin produced significantly greater MADRS reduction, confirming that its antidepressant efficacy exceeds that of an active placebo. Synthesizing this evidence, we note that psilocin’s antidepressant effect may involve parallel processes at the molecular, cellular, circuit, and experiential levels. However, because preclinical mechanisms were derived from rodents lacking the psychotherapeutic context and subjective dimensions of human trials, the following sections present these levels of evidence separately and highlight only putative—rather than established—connections between them. PSI’s “single-dose, sustained efficacy” in clinical trials appears consistent with—but does not establish—the rapid plasticity induction observed in preclinical models, suggesting potential intervention pathways for patients with TRD that remain to be validated independently of traditional monoaminergic pharmacotherapy paradigms.

Putative links exist between 5-HT_2a_ receptor actions and cortical network changes. At the preclinical level, Huang et al. used AAV-mediated shRNA knockdown to identify Htr2a in OFC layer-5 pyramidal neurons as necessary for psilocin-induced behavioral changes in mice [34], while Torrado Pacheco et al. showed that psilocin enhances cognitive flexibility in rats via 5-HT_2a_ receptors [22]. At the clinical level, Daws et al. reported reduced brain network modularity and increased executive network flexibility following psilocybin therapy in patients with depression [40], while Shukuroglou et al. observed attenuated ventral striatum–DMN connectivity [37], and Goodwin et al. described dose-dependent associations between psilocybin exposure, subjective experience, and functional connectivity changes [45]. The pharmacological specificity of such network-level effects is further supported by an active placebo-controlled trial showing that niacin, despite producing noticeable somatic effects, failed to match psilocybin’s antidepressant efficacy [66]. Although it is tempting to infer that OFC pyramidal 5-HT_2a_ activation in rodents directly translates to the network demodularization seen in human fMRI studies, such a cross-scale causal chain remains hypothetical. The human DMN and executive networks differ substantially in organization from rodent homologs, and the psychotherapeutic context present in all clinical trials is absent from animal models. Therefore, while rodent data generate testable hypotheses about 5-HT_2a_-mediated plasticity, they do not provide direct mechanistic explanations for clinical network reorganization. Erkizia-Santamaría et al. observed that psilocin reverses anhedonia and upregulates cortical 5-HT_2a_ receptor expression in CUMS mice [23], and Wojtas et al. reported dose-dependent bidirectional regulation of hippocampal 5-HT_2a_ receptors alongside increased nucleus accumbens monoamine release [25]. These findings illustrate receptor-level dynamics in rodents that may inform, but do not establish, dose–effect relationships observed in human trials.

BDNF-TrkB Plasticity: From rodent synapses to sustained clinical response. Preclinical studies by Wang et al. showed that early psilocybin intervention restores BDNF and TrkB levels and activates ERK, Akt, and mTOR phosphorylation in stress-sensitive Wistar rats and in WKY rats modeling treatment-resistant features [28,29]. Kolasa et al. further reported that psilocin upregulates neurotrophin-4 (Ntf4) mRNA in WKY rats [30]. These findings demonstrate rapid synaptic plasticity signaling in rodents. Zhao et al. bridged this signaling cascade to structural endpoints, showing that in a glucocorticoid stress model, BDNF-TrkB-mTOR rescue occurs concurrently with dendritic spine restoration and hippocampal neurogenesis, supporting the hypothesis that rapid molecular changes and anatomical plasticity may be coupled rather than sequential events [31]. In parallel, clinical trials report sustained antidepressant effects lasting weeks to months after single or limited psilocybin doses [42,47,59]. Notably, Gukasyan et al. extended this observation to 12 months in MDD using a two-dose regimen, documenting sustained response and remission rates of 75% and 58%, respectively, further supporting the durability of multi-dose PSI-assisted therapy [62]. Erritzoe et al. noted that at 6 months, psilocybin and escitalopram produced comparable depression score improvements, yet the psilocybin group showed greater gains in work and social functioning, connectedness, and life meaning [50]. Whether this divergence between symptom relief and functional recovery reflects BDNF-TrkB-mediated structural remodeling in humans—as hypothesized from animal data—cannot be determined from the current evidence. The absence of longitudinal synaptic biomarkers in human trials, along with the lack of psychotherapeutic context in animal models, precludes direct mechanistic attribution. Kolasa et al.’s observation of strain-dependent Ntrk2 expression and psilocin-response heterogeneity [30] raises the hypothesis that BDNF-system variation might contribute to individual differences in clinical response—an idea that requires direct testing in human pharmacogenetic studies rather than inference from rodent strain comparisons.

Amygdala–Prefrontal Interactions: Preclinical activation and clinical emotional processing. In rodents, Funk et al. reported dose-dependent activation of CeA and mPFC neurons following psilocybin, with prominent CeA engagement [32], while García-Cabrerizo et al. linked hippocampal NeuroD1^+^ cell increases to behavioral improvement [33]. In humans, Wall et al. found enhanced superior temporal gyrus responses to music after psilocybin therapy [38], Harding et al. reported improved anhedonia and preserved music-evoked vitality [39], Henry et al. noted improved optimism and dysfunctional attitudes [55], and Barba et al. observed reduced rumination and thought suppression [51]. Although these rodent and human findings appear conceptually aligned, direct cross-species mapping is problematic. Rodents lack the capacity to verbally report the mystical experiences, ego dissolution, or emotional breakthroughs that Weiss et al. identified as mediators of psilocybin’s superior efficacy over escitalopram [55]; consequently, animal models cannot validate the subjective mechanisms central to human psychedelic therapy. Furthermore, while Thomas et al. showed that psilocin alters prefrontal sleep–wake architecture in mice [35] and Liu et al. reported enhanced cingulate–prefrontal–striatal–hippocampal connectivity in rats [36], the default mode network architecture in rodents differs markedly from that in humans, limiting the validity of cross-species inferences about fMRI connectivity changes [37,38].

Multi-Receptor Profiles: Implications for differential clinical effects. Preclinical studies indicate that psilocin’s behavioral effects involve not only 5-HT_2a_ but also 5-HT_1β_ receptors [26], require intact 5-HTT function [27], and may include 5-HT_2a_-independent pathways [24]. Clinically, psilocybin showed superior response and remission rates vs. escitalopram in some measures [58], reduced experiential avoidance [52], and improved rumination and thought suppression [51], whereas escitalopram produced more limited cognitive effects [48,53]. Whether these clinical differences stem from psilocin’s multi-receptor pharmacology—as opposed to the single-target SSRI mechanism—remains speculative. The psychotherapeutic framework surrounding psilocybin administration confounds attempts to attribute clinical outcomes purely to receptor pharmacology, and animal behavioral endpoints lack equivalents for human constructs such as “life meaning” or “psychological flexibility”. Luquiens et al. reported that psilocybin improved depressive symptoms and alcohol cravings in comorbid patients [60]—an effect potentially attributable to its broad receptor profile, although direct translation from rodent receptor studies to human dual-diagnosis outcomes is not currently supported. Furthermore, a post hoc analysis of the Carhart-Harris et al. [48] trial demonstrated that prior SSRI/SNRI discontinuation abolished PSI’s superiority over escitalopram, with unmedicated patients showing a robust advantage of PSI that was absent in discontinuers [49]. This finding suggests that chronic serotonergic medication use and its downstream receptor adaptations—such as 5-HT_2a_ receptor downregulation—may constitute an important moderator of PSI’s antidepressant efficacy, independent of the acute subjective experience.

Despite these putative parallels, several fundamental gaps hinder the direct translatability of preclinical findings to human clinical outcomes. First, rodent behavioral assays (e.g., the forced swim test, the sucrose preference test) measure acute stress responses under controlled conditions and do not capture the chronicity, recurrence, cognitive–emotional complexity, or social impairment characteristic of human depression. Second, pharmacokinetic parameters differ markedly across species: psilocybin is metabolized more rapidly in rodents, and the doses used (e.g., 0.3–10 mg/kg) lack standardized human equivalents, raising the possibility of non-specific effects. Third, all human efficacy trials embedded psilocybin within extensive psychotherapeutic support (preparation, monitoring, integration)—a contextual factor entirely absent from animal studies that contributes independently to outcomes through expectancy and therapeutic alliance. Fourth, subjective experiences such as mystical states and ego dissolution—identified clinically as key mediators of antidepressant response—have no parallel in rodent models, leaving a central mechanism of human psychedelic therapy unaddressed preclinically. Fifth, although homologous brain regions exist, the default mode network and large-scale network architecture in rodents differ sufficiently from those in humans, cautioning against direct cross-species inference of fMRI connectivity changes. Finally, animal models used to predict antidepressant activity (e.g., CUMS, WKY) capture only specific etiological pathways, whereas human treatment-resistant depression arises from heterogeneous genetic, immunological, and environmental factors. Consequently, the preclinical evidence reviewed here should be interpreted as hypothesis-generating rather than as definitive mechanistic explanations for the clinical efficacy of psilocybin-assisted therapy.

#### 3.6.2. Limitations of Existing Evidence and Challenges in Clinical Translation

Although this review has systematically synthesized the multilevel evidence for the antidepressant effects of PSI, psilocybin still faces multiple challenges in clinical translation. First, the safety evidence for PSI remains insufficient; existing studies have demonstrated that it can induce psychological adverse reactions such as hallucinations, fear, anxiety, sleep disturbances, new-onset or exacerbated manic symptoms, and hallucinogen persisting perception disorder [95], as well as physiological adverse reactions including headache, dizziness, and nausea [96,97]. Second, the contraindications for specific populations remain unclear. On the one hand, pregnant and lactating women face potential biological risks and should be regarded as a special population requiring focused attention at the current stage. Animal studies have shown that PSI-treated dams exhibit increased anxiety-like behaviors, and offspring exposed to psilocybin through breast milk display anhedonia in adulthood, suggesting that PSI may not only pose potential long-term risks to pregnant and lactating women themselves but may also cross the blood–milk barrier to exert distal effects on the neurodevelopment of offspring [98]. On the other hand, for psychiatrically susceptible populations such as those with a family history of schizophrenia or BD, whether PSI should be considered an absolute contraindication still lacks clear criteria, and more safety data are urgently needed to define its risk–benefit boundary [99,100]. Third, the potential drug–drug interactions between PSI and specific medications have not been adequately evaluated. Studies have shown that the combination of PSI with monoamine oxidase inhibitors (MAOIs) may induce severe adverse reactions such as hypertensive crisis [101], and antidepressants such as SSRIs may attenuate the acute subjective effects of PSI [102]. Furthermore, the methodological quality of the included studies tempers the certainty of the evidence. While the 17 RCTs demonstrated robust data integrity, their overall risk of bias was uniformly rated as “some concerns” due to challenges in randomization and blinding. The 15 non-randomized studies exhibited a predominantly serious risk of bias, particularly in confounding control and outcome measurement, and the 15 animal studies were limited by unclear allocation concealment and inadequate caregiver blinding. These methodologic gaps underscore the need for cautious interpretation of mechanistic and efficacy findings. Finally, the role of psychotherapy in the observed clinical efficacy must be explicitly acknowledged. Virtually all clinical trials of psilocybin in depression conducted to date have embedded the compound within an extensive psychotherapeutic framework, comprising preparatory sessions, continuous professional support during dosing, and post-session integration therapy. Consequently, the efficacy synthesized in this review cannot be attributed to the pharmacological action of psilocybin alone but rather reflects the combined effect of the drug administered within a structured psychotherapeutic context. Psychological support may contribute to treatment outcomes through expectancy modulation, therapeutic alliance, and the facilitation of consolidation of psychological insight and emotional breakthrough experiences during integration sessions. Because none of the included trials employed a factorial design isolating the pharmacological effect from the psychotherapeutic effect, the independent contribution of each component cannot be quantified from the current evidence. Although the contribution of psychotherapy is more difficult to ascertain at the neurobiological level, future trials should adopt designs capable of disentangling these components—such as 2 × 2 factorial designs comparing psilocybin vs. placebo, with or without standardized psychotherapy—and report psychotherapeutic components in sufficient detail to permit cross-trial comparison. These limitations indicate that the clinical translation of PSI necessitates further high-quality research to optimize its clinical translation.

#### 3.6.3. Future Prospects

The future development of PSI antidepressant therapy needs to overcome three major bottlenecks: insufficient safety evidence, unclear contraindications for special populations, and inadequately assessed drug interaction risks. First, a standardized adverse event reporting and monitoring system should be established. Given that current clinical trials employ inconsistent standards for documenting adverse reactions, there is an urgent need to develop a dedicated grading scale for serious adverse events in psychedelic therapy, as well as a long-term follow-up registry system. Through multicenter collaborative networks, researchers should systematically collect data on the incidence, duration, and outcomes of common adverse reactions such as headache, nausea, anxiety, and blood pressure fluctuations, while simultaneously tracking rare but severe events including suicidal ideation, mania induction, and persistent perceptual disorders. Second, safety studies targeting pregnant and lactating women should be conducted. Systematic reproductive toxicity assessments should be performed in non-human primates to clarify the transfer rate of PSI across the blood–milk barrier and the level of exposure. Before obtaining sufficient preclinical safety data, pregnant and lactating women should be explicitly excluded from PSI clinical trials, and a pregnancy exposure registry system should be established to collect long-term follow-up data from accidental exposure cases. Furthermore, the risk–benefit profile for psychiatrically susceptible populations should be defined. For individuals with a family history of schizophrenia or bipolar disorder, multicenter risk-stratification studies are recommended, combining polygenic risk scores with baseline brain functional connectivity features to prospectively evaluate the incidence of psychiatric adverse events following PSI administration in this population. On this basis, inclusion and exclusion criteria based on individual risk stratification should be formulated (rather than blanket exclusion), thereby safeguarding patient safety while preserving opportunities for the future expansion of potential indications. Finally, the interaction spectrum between PSI and commonly used psychiatric medications should be systematically evaluated. Clinical pharmacology study designs should be adopted to systematically investigate the pharmacokinetic and pharmacodynamic interactions of PSI when co-administered with MAOIs, SSRIs, SNRIs, and benzodiazepines.

Future research may also integrate neuroimaging biomarkers, peripheral blood inflammatory indicators, and genetic polymorphism information to construct multivariate models for predicting PSI efficacy. Through efficacy difference analyses across different endotype subgroups, the patient populations most likely to benefit can be screened, thereby achieving the transition of PSI toward biomarker-based individualized treatment. In this regard, Dougherty et al. have provided proof-of-concept evidence that natural language processing of post-dosing integration sessions can predict treatment response in TRD with high accuracy [103], pointing toward linguistic biomarkers as readily accessible tools for personalized psychedelic therapy.

## 4. Conclusions

In summary, this systematic review synthesizes 46 original studies published within the past five years, moving beyond previous fragmented mechanistic reports by integrating critical evidence chains from psilocin’s action mechanisms to clinical efficacy. By systematically delineating psilocin’s effects from molecular targets through clinical outcomes, this review identifies convergent evidence and critical translational gaps—particularly the lack of validated cross-species behavioral endpoints and the absence of mechanistic biomarkers in human trials. These gaps define priorities for future research. The compiled evidence suggests that psilocin may alleviate core depressive symptoms via multilevel mechanisms involving multi-receptor action, synaptic plasticity remodeling, and reorganization of corticolimbic circuits, providing a potential novel intervention framework beyond traditional monoaminergic pharmacotherapy paradigms. Based on the current evidence, PSI offers an effective novel therapeutic option for patients with depression that transcends the traditional monoaminergic pharmacotherapy paradigm, contingent upon controlled medical settings, rigorous patient screening, and professional psychological support. However, a significant gap remains between research evidence and routine clinical application. Currently, limitations in long-term follow-up data and the scarcity of safety information for susceptible populations are insufficient to support PSI’s broad implementation outside clinical trial settings. Future research urgently requires multicenter, large-sample, long-duration randomized controlled trials; only upon resolving the aforementioned safety and standardization issues can PSI be robustly translated from laboratory evidence to clinical practice.

## Figures and Tables

**Table 1:** Characteristics and main findings of included animal experimental studies on psilocybin’s antidepressant mechanisms at the molecular, cellular, and neural circuit levels.

Author	Sample	Intervention/Dose	Intervention Duration	Outcome Measures	Main Findings
Torrado Pacheco et al. [22]	49 Long–Evans rats (35 completed)	PSI 1 mg/kg, i.p.	Acute (single injection)	Set-shifting task (cognitive flexibility)	PSI selectively enhanced set-shifting ability, at least partially through 5-HT_2a_ receptor activation, suggesting improved cognitive flexibility as a potential adjunctive mechanism for antidepressant therapy.
Erkizia-Santamaría et al. [23]	64 male C57BL/6J mice, CUMS model	PSI 1 mg/kg, i.p. ×2 injections	Acute (two injections, 7 days apart)	Anhedonia (sucrose preference), behavioral despair (TST, FST), anxiety-like behavior (EPM, OFT, NSFT), apathy (NBT, coat state), HTR; cortical gene and protein expression	PSI reversed CUMS-induced anhedonia and behavioral despair (TST, FST), and exerted anxiolytic-like effects, but did not rescue apathy-related behaviors (NBT, coat state scores) or HPA-axis hyperactivity (adrenal hypertrophy). PSI increased cortical 5-HT_2a_ receptor expression and function (enhanced HTR after booster dose) and selectively upregulated glucocorticoid receptor (Nr3c1) mRNA in CUMS mice, yet it failed to reverse stress-induced decreases in cortical BDNF protein levels.
Hesselgrave et al. [24]	Chronically stressed male C57BL/6J mice	PSI 10 mg/kg, i.p.	Acute (single injection)	Anhedonia (sucrose preference), hippocampal AMPA/NMDA ratio (synaptic strength), head-twitch response	PSI reversed chronic stress-induced anhedonia and enhanced hippocampal TA-CA1 and synaptic AMPA/NMDA ratios; these effects were not blocked by the 5-HT_2a_/_2_c antagonist ketanserin, suggesting a 5-HT_2a_-receptor-independent antidepressant mechanism.
Wojtas et al. [25]	Adult male Wistar-Han rats	PSI 2 mg/kg and 10 mg/kg, s.c.	Acute (single injection)	Brain neurotransmitters (DA, 5-HT, Glu, GABA, ACh), receptor density (5-HT1A, 5-HT2A, D2), anxiety-like behavior (EPM, OFT)	PSI dose-dependently bidirectionally regulated hippocampal 5-HT_2_A receptors (down at 2 mg/kg, up at 10 mg/kg) as a lasting effect, while both doses elevated accumbal DA and 5-HT release and exerted anxiolytic-like effects in naive rats.
Fleury et al. [26]	5-HT1BR-knockout and littermate control mice, male and female	PSI 5 mg/kg, i.p.	Acute (single injection); longitudinal behavioral follow-up	Head-twitch, locomotion, anhedonia (gustometer), anxiety-like behavior (EPM, NSF, OFT), c-Fos whole-brain analysis	PSI significantly reversed stress-induced sucrose licking reduction (anhedonia) in mice—an effect abolished in 5-HT_1_B-receptor-knockout mice under specific stress paradigms—establishing 5-HT_1_B receptors as essential molecular substrates for psilocybin’s antidepressant-like effects in mice.
Gattuso et al. [27]	5-HTT-knockout (KO) and wild-type (WT) C57BL/6J mice, male and female	PSI 1 mg/kg, i.p.	Acute (single injection)	Head-twitch response, locomotor activity, Porsolt swim test (immobility), anxiety-like behavior (light–dark box)	PSI showed no significant antidepressant-like effect in the Porsolt swim test across genotypes (with only a delayed trend in WT females), while its acute hyperlocomotor and head-twitch effects were entirely dependent on 5-HTT functional integrity, as they were absent in 5-HTT-knockout mice.
Wang et al. [28]	22 male Wistar rats, chronic social instability stress (SIS) + predator odor exposure (POE)	PSI 1.0 mg/kg, i.p.	Early intervention (1 h post-stress/POE)	OFT, SPT, NORT, EPM, FST; serum BDNF; brain BDNF/TrkB/pERK/pAkt/pmTOR in PFC, AMG, HYP, and HIP	Early PSI intervention alleviated chronic stress-induced behavioral despair and cognitive deficits via BDNF-TrkB-mTOR-dependent neuroplasticity restoration and endocannabinoid-system-mediated HPA-axis modulation.
Wang et al. [29]	22 male Wistar-Kyoto (WKY) rats, SIS + POE, TRD-like paradigm	PSI 1.0 mg/kg, i.p.	Early intervention (1 h post-POE)	OFT, SPT, NORT, EPM, FST; serum BDNF; brain CB1R, BDNF, TrkB, pERK, pAkt, pmTOR; 2-AG levels	In WKY rats, early PSI alleviated stress-induced behavioral despair and cognitive impairment. Mechanistically, PSI restored suppressed TSH levels and reversed BDNF/TrkB deficits with region-specific ERK/Akt/mTOR activation, while upregulating CB1R without altering 2-AG, indicating ECS modulation distinct from non-TRD stress paradigms.
Kolasa et al. [30]	Male Wistar-Han (7 wk, ~220 g) and Wistar-Kyoto (7 wk, ~200 g) rats	PSI 0.3 mg/kg, i.p.	Acute (single injection); behavioral testing at 4 h, 6 d (NORT), 2 d, 8 d (SI), 9 d, and 23 d (FST)	NORT, social interaction, FST; prefrontal cortex gene expression (BDNF, TrkB, NT3, NT4, Arc, Gria1, PSD95, St8sia2, St8sia4); serum BDNF	In Wistar-Kyoto (WKY) rats, a single low-dose PSI (0.3 mg/kg) administration significantly upregulated Arc mRNA and protein expression in the prefrontal cortex, alongside a main effect on BDNF mRNA expression and specific upregulation of neurotrophin-4 (Ntf4) mRNA, suggesting strain-specific transcriptional regulation of neuroplasticity-related genes in this TRD-like paradigm. Notably, serum BDNF and cortical BDNF protein levels remained unchanged at the 24-day timepoint.
Zhao et al. [31]	Male C57BL/6J mice, chronic CORT exposure	PSI 0.1–2.5 mg/kg, i.p.	Acute (single dose)	FST, SPT, NSFT; Golgi staining; WB; immunofluorescence	PSI (2.5 mg/kg) rapidly and sustainably reversed CORT-induced depressive-like behaviors, restored dendritic complexity and spine density in the PFC and hippocampus, and rescued synaptic protein levels and BDNF-TrkB-mTOR signaling activation.
Funk et al. [32]	Male Sprague Dawley rats (*n* = 32)	PSI 0.1–3 mg/kg, s.c.	Single dose	Fos protein expression (IHC) in mPFC, NAc, CeA, BLA, LC, and DRN	PSI dose-dependently increased Fos expression in multiple brain regions, including the mPFC, nucleus accumbens shell, and locus coeruleus, with the most pronounced effect observed in the central amygdala (CeA), where approximately 50% of neurons were activated by the high dose (3 mg/kg), compared with approximately 10% in controls. These findings, along with the observation that PSI also activated oligodendrocytes (Olig1+), suggest that acute CeA activation may represent a key initiating event for psilocybin’s sustained therapeutic effects, rather than a directly established antidepressant mechanism, warranting further functional validation.
García-Cabrerizo et al. [33]	Male and female Sprague Dawley rats, adolescents (PND 34–46)	PSI 0.3 and 1 mg/kg, oral gavage	Repeated: 7 days, once daily	Hippocampal neurogenesis markers (Ki-67, NeuroD1, BrdU); FST, TST, sucrose preference	The 1 mg/kg PSI dose significantly increased hippocampal NeuroD1^+^ progenitor cell numbers, which showed a significant correlation with long-term antidepressant-like behavioral improvements, suggesting the promotion of hippocampal neurogenesis as a potential cellular plasticity mechanism and biomarker.
Huang et al. [34]	Male C57BL/6J mice (*n* = 33)	PSI 1 mg/kg, i.p.	Single dose	Cell-type-specific neuronal changes in OFC (snRNA-seq, electrophysiology, behavior)	Knockdown of Htr2a in OFC layer-5 pyramidal neurons abolished the antidepressant-like effects of PSI and blocked PSI-induced GluR1 downregulation, while knockdown in PV neurons only partially attenuated these effects, establishing OFC layer-5 pyramidal neurons and Htr2a as essential cellular targets for PSI action.
Thomas et al. [35]	Male C57BL/6J mice (*n* = 8)	Psilocin 0.25 mg/mL → 2 mg/kg, i.p.	Single dose; with/without sleep deprivation	Sleep–wake architecture, cortical LFP/EEG spectral power, homeostatic rebound	Acute psilocin disrupted sleep maintenance and slowed the local dissipation of sleep pressure (slow-wave activity) specifically in prefrontal cortical LFPs, without affecting global EEG sleep homeostasis, suggesting region-specific modulation of local sleep regulation.
Liu et al. [36]	Male rats (*n* = 15)	Psilocin hydrochloride 2.0 mg/kg, i.p.	Single dose; acute BOLD fMRI + EGR1 IHC	BOLD fMRI functional connectivity; EGR1 expression across cortical/striatal regions	Psilocin (2.0 mg/kg) increased functional connectivity between the cingulate cortex and prefrontal cortex, striatum, and hippocampus, while decreasing connectivity with the septum, amygdala, thalamus, and substantia nigra. These changes were accompanied by a mixed pattern of cortical/subcortical activity alterations and widespread EGR1 upregulation, indicating psilocin-induced hyperactivity across corticostriatal networks.

**Table 2:** Characteristics and main findings of included clinical neuroimaging studies on psilocybin’s neural circuit mechanisms in depression.

Author	Participants	Intervention/Dose	Intervention Duration	Outcome Measures	Main Findings
Shukuroglou et al. [37]	Patients with TRD (*n* = 19)	PSI 10 mg and 25 mg, oral	Two sessions, 1 week apart; follow-up at 1 day, 1 week, and 3 months	Music-evoked emotion (pleasantness ratings); NAc-DMN functional connectivity (fMRI)	PSI therapy for treatment-resistant depression attenuated functional connectivity between the nucleus accumbens and the default mode network (DMN) while listening to music.
Wall et al. [38]	Patients with TRD (*n* = 19)	PSI 10 mg and 25 mg, oral	Two sessions, 1 week apart; fMRI at 1 week pre- and 1 day post-treatment	Low-frequency brain responses to music (ALFF fMRI)	PSI therapy enhanced bilateral STG ALFF while listening to music, as well as reducing medial frontal ALFF during rest, indicating region- and state-specific modulation of low-frequency neural activity.
Harding et al. [39]	41 patients with MDD (PSI *n* = 22; escitalopram *n* = 19)	PSI 25 mg × 2 vs. escitalopram 20 mg/day	Two sessions, 3 weeks apart; 6-week follow-up	Musical surprise processing (fMRI ROI activation, subjective valence/arousal ratings)	PSI therapy reduced vmPFC responses and increased sensory cortical responses to musical surprises, while escitalopram relatively enhanced default-mode/associative region activation, indicating divergent neural processing modes that may reflect reduced top-down predictive precision with PSI therapy vs. enhanced cognitive/attentional processing with escitalopram.
Daws et al. [40]	TRD *n* = 16; MDD *n* = 43 (RCT)	PSI 25 mg × 2 vs. escitalopram	Two sessions, 1 week (open-label) or 3 weeks (RCT) apart; 6-week to 6-month follow-up	Brain network modularity, fMRI global integration, BDI depression scores	PSI therapy reduced whole-brain network modularity and increased DMN-EN/SN cross-network integration, with no such alterations observed with escitalopram.

**Table 3:** Characteristics and main findings of included clinical studies on psilocybin’s antidepressant efficacy.

Author	Participants	Intervention/Dose	Intervention Duration	Outcome Measures	Main Findings
Ellis et al. [41]	US military veterans with severe TRD (*n* = 15)	Single-dose 25 mg PSI with psychological support	Single dose; 6–8 h session; 12-week follow-up	MADRS, QIDS, SDS, C-SSRS, 5D-ASC	A single dose of 25 mg achieved 60% response and 53% remission at week 3, with 47% response and 40% remission sustained at week 12. Mean MADRS reduction was −23.00 (Cohen’s d = 2.23). Comorbid PTSD did not moderate outcomes, and 5D-ASC scores were not correlated with response. No serious adverse events occurred.
Ellis et al. [42]	US military veterans with severe TRD (*n* = 15)	Single-dose 25 mg PSI with psychological support	Single dose; 12-month follow-up	MADRS, QIDS, SDS, 5D-ASC	Month 6: 80% response, 50% remission. Month 12: 40% response, 30% remission. Effects waned after month 6 but remained significantly below baseline (54% MADRS reduction). No serious adverse events.
Goodwin et al. [43]	TRD (n = 233: 25 mg *n* = 79, 10 mg n = 75, 1 mg *n* = 79)	Single dose of 25 mg, 10 mg, or 1 mg COMP360 PSI + psychological support	Single dose; 12-week follow-up	MADRS, QIDS-SR, PANAS, WSAS	25 mg significantly reduced MADRS compared with 1 mg at week 3 (−12.0 vs. −5.4; *p* < 0.001), with 37% response and 29% remission; 10 mg showed no significant difference (*p* = 0.18). Adverse events occurred in 77% of participants, including headache, nausea, and dizziness; suicidal ideation or behavior was reported across all dose groups.
Goodwin et al. [44]	TRD (*n* = 233: 25 mg *n* = 79, 10 mg *n* = 75, 1 mg *n* = 79)	Single dose of 25 mg, 10 mg, or 1 mg COMP360 PSI + psychological support	Single dose; 12-week follow-up	QIDS-SR-16, GAD-7, PANAS, WSAS	25 mg improved patient-reported depression (QIDS-SR-16: −2.8 vs. 1 mg), anxiety (GAD-7: −1.8), affect (PANAS positive: +6.2; negative: −3.2), and function (SDS: −6.5; WSAS: −5.1) at week 3 compared with 1 mg, with a dose–response relationship; 10 mg showed smaller effects.
Goodwin et al. [45]	TRD (*n* = 233)	Single dose of 25 mg, 10 mg, or 1 mg COMP360 PSI + psychological support	Single dose; 12-week follow-up	MADRS, 5D-ASC, EBI, dose–response	Psychedelic effects were dose-dependent yet exhibited substantial overlap across doses. Depression improvement correlated with oceanic boundlessness (r = −0.508), visual restructuralization (r = −0.516), and EBI (r = −0.637) at 25 mg but not anxious ego dissolution, supporting specific experiential domains as pharmacodynamic biomarkers of response.
Kirlić et al. [46]	TRD (*n* = 233)	Single dose of 25 mg, 10 mg, or 1 mg COMP360 PSI	Single dose; post-session acute assessment	5D-ASC, EBI, pretreatment clinical characteristics	Dose was the strongest predictor of psychedelic intensity; pretreatment positive affect, lower anxiety, higher executive function, and personality disorder symptoms weakly but significantly modulated specific 5D-ASC/EBI dimensions.
Goodwin et al. [47]	TRD (*n* = 66; 25 mg *n* = 22, 10 mg *n* = 19, 1 mg *n* = 17 from COMP 001; 8 from COMP 003)	Single dose of 25 mg, 10 mg, or 1 mg COMP360 PSI	Single dose; 52-week follow-up	Time to depressive event, AEs	The 25 mg dose was associated with a longer median time to depressive event (92 days) compared with the 10 mg (83 days) and 1 mg (62 days) doses; post hoc enrolled analysis showed 189 vs. 43 vs. 21 days. Few AEs occurred after week 12.
Carhart-Harris et al. [48]	59 patients with MDD (PSI *n* = 30; escitalopram *n* = 29)	PSI 25 mg × 2 + placebo vs. escitalopram 10–20 mg/day	Two sessions, 3 weeks apart; 6-week follow-up	QIDS-SR-16, QIDS-SR-14, QIDS-SR-16 response/remission, BDI-IA, MADRS, HAM-D-17, safety/adverse events	No significant difference in primary QIDS-SR-16 at week 6 (−8.0 vs. −6.0; *p* = 0.17); secondary outcomes favored psilocybin (70% response, 57% remission vs. 48%, 28%). Similar adverse event rates; no serious AEs.
Erritzoe et al. [49]	59 patients with MDD (PSI *n* = 30; escitalopram *n* = 29)	PSI 25 mg × 2, oral + psychological support vs. escitalopram 10–20 mg/day + PSI 1 mg × 2 (placebo control) + matched psychological support	Two sessions, 3 weeks apart; 6-week trial period with final follow-up	Depression severity (QIDS-SR-16, BDI, HAM-D, MADRS), psychological well-being (WEMWBS), acute psychedelic experience (MEQ, EDI, EBI, CEQ), baseline expectation ratings	Participants who discontinued treatment (n = 21) exhibited increased depression severity from screening to baseline (QIDS-SR-16 β = 2.21, 95% CI 0.21–4.23; BDI β = 3.66, 95% CI 0.85–6.48)—a pattern consistent with withdrawal symptoms. Among unmedicated patients (n = 35), PSI significantly outperformed escitalopram across all depression measures (QIDS-SR-16, BDI, HAM-D, MADRS; all *p* < 0.001) and well-being (WEMWBS; *p* < 0.001), yielding an additional 50–120% antidepressant effect; among discontinuers, no significant between-treatment differences were observed (all *p* > 0.05). Acute psychedelic experiences (MEQ, EDI, EBI, CEQ) were unaffected by discontinuation status (all *p* > 0.05). Response/remission rates and drop-out rates were not stratified by discontinuation status in this post hoc analysis; the parent trial reported overall response rates of 70% (PSI) vs. 48% (escitalopram) and remission rates of 57% vs. 28%.
Erritzoe et al. [50]	59 patients with MDD (PSI *n* = 30; escitalopram *n* = 29)	PSI 25 mg × 2 + placebo vs. escitalopram 10–20 mg/day	Two sessions, 3 weeks apart; 6-month follow-up	QIDS-SR-16, BDI, WSAS, WCS, MLQ at 6 weeks and 6 months; safety/adverse events	Both conditions showed sustained QIDS-SR-16 improvements at 6 months, with no significant difference (mean difference 1.51, *p* = 0.311); PT was superior in functioning (WSAS: −7.46, *p* < 0.001), connectedness (WCS: 11.02, *p* = 0.033), and meaning in life (MLQ: 4.86, *p* = 0.021).
Barba et al. [51]	59 patients with MDD (PSI *n* = 30; escitalopram *n* = 29)	PSI 25 mg × 2 vs. escitalopram 10–20 mg/day	Two sessions, 3 weeks apart; 6-week follow-up	Rumination (RRS), thought suppression (WBSI), depression severity (QIDS-SR-16, BDI)	PSI significantly reduced rumination (RRS: *p* < 0.001, d = 0.63) and thought suppression (WBSI: *p* < 0.001, d = 0.87) at 6 weeks compared with escitalopram (time × condition: *p* = 0.037 and *p* = 0.019); WBSI reduction was exclusive to PSI responders (70% response rate) vs. escitalopram responders (48%). Drop-out: 3 PSI and 4 escitalopram participants; remission rates were not reported.
Zeifman et al. [52]	59 patients with MDD (PSI *n* = 30; escitalopram *n* = 29)	PSI 25 mg × 2 + placebo vs. escitalopram 10–20 mg/day	Two sessions, 3 weeks apart; 6-week follow-up	Experiential avoidance (BEAQ), depression severity, suicidal ideation, trait anxiety, connectedness (WCS), psychological insight	PSI, not escitalopram, significantly improved mental health via reduced experiential avoidance (all indirect effects *p* ≤ 0.015); significant between-group difference (*p* = 0.011–0.043). Drop-out: 3 vs. 4 participants missed second dosing or discontinued capsules; all 59 completed 6-week assessment. Response and remission rates were not reported.
Harding et al. [39]	41 patients with MDD (PSI *n* = 22; escitalopram *n* = 19)	PSI 25 mg × 2 vs. escitalopram 20 mg/day	Two sessions, 3 weeks apart; 6-week follow-up	Anhedonia (SHAPS), music-evoked emotions (GEMS-25: vitality, sublimity, unease), surprise-related valence/arousal ratings	PT reduced anhedonia more than escitalopram (*p* = 0.048). Escitalopram decreased music-evoked vitality (*p* = 0.023) and abolished surprise-related valence (*p* = 0.220), whereas PT preserved the latter (*p* = 0.001). Of 59 patients enrolled in the parent trial, 50 were initially included in this secondary analysis, with 9 lost due to incomplete datasets from COVID-19 lockdowns. A further 5 participants were excluded due to excessive fMRI motion or protocol deviations, leaving 41 patients with usable fMRI data in the final analysis.
Daws et al. [40]	TRD *n* = 16; MDD *n* = 43 (RCT)	PSI 25 mg × 2 vs. escitalopram	Two sessions, 1 week (open-label) or 3 weeks (RCT) apart; 6-week to 6-month follow-up	Depression severity (BDI-1A)	PSI therapy yielded rapid, sustained BDI score reductions in treatment-resistant depression at 1 week and 6 months (*p* < 0.001, d = 1.78 and 1.07 respectively), with superior BDI symptom reduction compared with escitalopram in major depressive disorder at weeks 2, 4, and 6 (arm × time interaction *p* = 0.005, all timepoints *p* ≤ 0.013, d = 0.75–0.98). Across the total 78 recruited participants, 11 were excluded for excessive fMRI head motion, and 8 discontinued intervention or were lost to follow-up; formal response and remission rates were not reported in this neuroimaging analysis.
Martens et al. [53]	59 patients with MDD (PSI *n* = 30; escitalopram *n* = 29)	PSI 25 mg × 2 vs. escitalopram 10–20 mg/day	Two sessions, 3 weeks apart; 6-week follow-up	FERT (accuracy, misclassifications, RT, response bias, target sensitivity), QIDS-SR-16	There were no significant between-group differences in negative affective bias reduction (p’s > 0.475). Longitudinally, decreased positive-to-negative misclassification correlated with reduced depression at week 10 in escitalopram only (r = 0.498, *p* = 0.025). Drop-out: 8 escitalopram (4 AEs, 4 lost to follow-up) and 6 PSI (4 lost, 1 cannabis use, 1 stopped placebo). Response and remission rates not reported in this secondary analysis.
Henry et al. [54]	59 patients with MDD (PSI *n* = 30; escitalopram *n* = 29)	PSI 25 mg × 2 vs. escitalopram 10–20 mg/day	Two sessions, 3 weeks apart; 6-week follow-up	DAS-24, LOT-R, POFLE, BDI-1A, FS	PSI increased optimism (LOT-R: +6.63, *p* < 0.0001, d = 1.1) and improved all DAS-24 domains (*p* < 0.0001) vs. escitalopram (achievement only, *p* = 0.005). Greater BDI-1A reduction (*p* = 0.011) and flourishing increase (*p* = 0.039) with PSI. Drop-out: 8 escitalopram (4 AEs, 4 lost) and 6 PSI.
Weiss et al. [55]	59 patients with MDD (PSI *n* = 30; escitalopram *n* = 29)	PSI 25 mg × 2 vs. escitalopram 10–20 mg/day	Two sessions, 3 weeks apart; 6-week follow-up	Depression factor score, MEQ, EDI, EBI, CEQ, music impact, intensity, MODTAS, MISS	Mystical experience and ego dissolution significantly mediated the effects of PT compared with ET on depression (MEQ mystical: −0.64, *p* < 0.001; ego dissolution: −0.49, *p* < 0.001). Emotional breakthrough and the impact of music moderated the response to PT (*p* ≤ 0.049). Drop-out: 8 escitalopram (4 AEs, 4 lost to follow-up) and 6 PSI. Response and remission rates not reported in this secondary analysis.
Weiss et al. [56]	59 patients with MDD (PSI *n* = 30; escitalopram *n* = 29)	PSI 25 mg × 2 vs. escitalopram 10–20 mg/day	Two sessions, 3 weeks apart; 6-week and 6-month follow-up	BFI, BFAS, MODTAS, BIS-B	No significant between-condition personality differences. PT decreased neuroticism, introversion, disagreeableness, and impulsivity and increased openness and absorption (*p* < 0.05). ET decreased neuroticism, disagreeableness, and impulsivity, and increased openness (*p* < 0.05). Drop-out: 8 escitalopram and 6 psilocybin. Response and remission rates not reported.
Rosenblat et al. [57]	TRD (*n* = 30, including 4 BDII and 26 MDD)	25 mg PSI + psychotherapy	Up to 3 flexible sessions; 2-week primary endpoint; 6-month total follow-up; 2-week primary endpoint	MADRS, QIDS-SR, GAD-7, CGI, C-SSRS	Significant MADRS reduction immediate vs. waitlist (−9.6 vs. −3; Hedge’s g = 1.07, *p* < 0.01); repeated doses administered as clinically indicated were associated with further incremental antidepressant benefit. Drop-out: 1 pre-dosing due to antidepressant tapering challenges, 1 before week-2 endpoint, 8 during 6-month follow-up to initiate other treatments; 21/30 total treated participants completed the full 6-month follow-up. Formal treatment-response/remission rates were not explicitly reported in this feasibility trial.
Szigeti et al. [58]	55 patients with MDD (PSI *n* = 29; escitalopram *n* = 26)	PSI 25 mg × 2 sessions vs. escitalopram 10–20 mg/day	Two sessions, 3 weeks apart; 6-week follow-up	QIDS-SR-16, HAM-D, BDI, MADRS, STAI-T, WEMWBS	Unadjusted models favored PSI on HAM-D, BDI, MADRS, and STAI-T (*p* ≤ 0.047). QIDS-SR-16 response 70% vs. 48%; remission 57% vs. 28%. Adjusted for baseline expectancy: between-group differences non-significant (all *p* > 0.05); adjusted for suggestibility: PSI superiority persisted (all *p* ≤ 0.047); n = 55 analyzed.
Agrawal et al. [59]	Patients with depression and cancer (*n* = 30)	Single-dose 25 mg PSI with psychological support	Single dose; 24-month follow-up	MADRS, HAM-A	At 24 months, 53.6% response (MADRS mean −15.0, *p* < 0.001) and 50% remission; 46.4% anxiety response (HAM-A mean −13.9, *p* < 0.001). Drop-out: 2 non-study-related deaths; 28/30 analyzed.
Luquiens et al. [60]	Alcohol-use disorder with comorbid depression (*n* = 30)	25 mg PSI (*n* = 20) vs. 1 mg (*n* = 10) + standard care	Two sessions, 3 weeks apart; 12-week follow-up	TLFB, CEQ, BDI-II, BAI, AQoLS	At 12 weeks, 25 mg significantly improved abstinence (55% vs. 11%; *p* = 0.043), percentage of drinking days (*p* = 0.038), and craving frequency (*p* = 0.045); BDI-II 80% response was 45% vs. 0% (*p* = 0.027). Drop-out: 1 in 25 mg group (unrelated MI) and 4 in 1 mg group (refused second dose). Relapse rates: 35% vs. 50% (non-significant).
Meikle et al. [61]	Treatment-resistant depression (*n* = 7)	Two 25 mg PSI sessions + 3 preparatory and 6 integration sessions	Two sessions, 4–6 weeks apart; 20-week follow-up	QIDS-SR, WHOQoL-Bref, GAD-7, BEAQ, WCS	Significant QIDS-SR reduction at 3 weeks (mean −7.14, *p* = 0.02, Hedges’ g = −1.27) and 20 weeks (mean −7.14, *p* = 0.02). Response and remission 43% (3/7). Drop-out: 1/8 withdrew pre-dosing (safety concern); 7 completed both doses. No serious adverse events.
Gukasyan et al. [62]	24 patients with moderate-to-severe MDD	PSI 20 mg/70 kg and 30 mg/70 kg, oral + supportive psychotherapy	Two sessions, 2 weeks apart; 12-month follow-up	GRID-HAMD, QIDS, BDI-II, MEQ30, Persisting Effects Questionnaire (well-being), C-SSRS, HPPD screening	Two doses of psilocybin produced large, sustained antidepressant effects through 12-month follow-up (GRID-HAMD Cohen’s d = 2.4, *p* < 0.001; response 75%, remission 58%). All 24 completers were retained through all follow-up visits (3 of 27 randomized did not complete both sessions). No serious adverse events, HPPD, suicidal ideation, or illicit psilocybin use occurred. Acute mystical and personally meaningful experiences predicted increased well-being but did not consistently predict sustained depression improvement beyond 4 weeks.
Aaronson et al. [63]	Severe TRD (*n* = 12)	Single-dose 25 mg synthetic PSI + psychological support	Single 8–9 h session; 12-week follow-up	MADRS, QIDS-SR-16, GAD-7, QLES-Q-SF, WSAS	Significant reductions in MADRS scores were observed at week 3 (LSM −15.8, *p* = 0.002) and week 12 (LSM −17.2, *p* = 0.0002). Response rates were 66.7% at week 3 and 58.3% at week 12, while remission rates were 41.7% and 25%, respectively. Drop-out: 1/12 withdrew before week 6; 11 completed. No serious AEs.
Calder et al. [64]	28 patients with treatment-resistant depressive and/or anxiety disorders	LSD 100–200 µg or PSI 15–25 mg per session; flexible multi-session regimen	Single session (~6–9 h); follow-up at mean 7.7 days	MADRS, real-time subjective effect ratings, MEQ	Significant MADRS reduction immediately (29%, *p* < 0.001) and at follow-up (*p* < 0.001). No dose–response (*p* = 0.83) or LSD vs. PSI difference (*p* = 0.43). Relaxation predicted improvement (*p* = 0.03). Drop-out, response, and remission rates were not reported in this naturalistic program. Most AEs were mild and resolved within 48 h.
Aboulafia-Brakha et al. [65]	115 patients (56.5% female; mean age 47.5) with treatment-resistant depressive/anxiety disorders	LSD 100 µg or PSI 25 mg per session (first fully active dose PAP session)	Single session (~8–9 h); 1–3-month follow-up	BDI, STAI-T, CERQ, MEQ	Significant BDI (F = 63.50, *p* < 0.001, η^2^ = 0.42) and STAI-T (F = 16.97, *p* < 0.001, η^2^ = 0.17) reductions; no LSD vs. PSI difference (*p* = 0.91); 37% achieved >50% BDI response. Drop-out: 0% treatment discontinuations. No serious AEs; adverse events mild and transient.
Raison et al. [66]	104 patients with MDD (PSI *n* = 51; niacin *n* = 53)	PSI 25 mg, oral + psychological support vs. niacin 100 mg + matched psychological support	Single dose; 6-week follow-up (assessments at days 2, 8, 15, 29, and 43)	MADRS, SDS, sustained response, AEs	A single 25 mg dose of PSI significantly reduced MADRS scores compared with niacin at day 43 (mean difference −12.3 [95% CI, −17.5 to −7.2] *p* < 0.001) and day 8 (mean difference −12.0 [95% CI, −16.6 to −7.4]; *p* < 0.001) and resulted in greater functional improvement (SDS: −2.31; *p* < 0.001). Sustained response was higher (42% vs. 11%; *p* = 0.002); remission showed a numerical advantage (25% vs. 9%; *p* = 0.05). Drop-out by day 43 was 2% (1/51) for PSI vs. 17% (9/53) for niacin. No serious treatment-emergent adverse events occurred; PSI was associated with higher rates of mild-to-moderate acute AEs (headache, nausea).

**Table 4:** Risk-of-bias assessment of included animal experimental studies using the SYRCLE tool.

Study	Q1	Q2	Q3	Q4	Q5	Q6	Q7	Q8	Q9	Q10
Torrado Pacheco et al. [22]	L	L	U	U	U	U	L	L	L	L
Erkizia-Santamaría et al. [23]	L	L	U	U	U	U	L	L	L	H
Wojtas et al. [25]	U	L	U	U	U	U	U	L	L	H
Hesselgrave et al. [24]	L	L	U	U	U	U	L	L	L	H
Fleury et al. [26]	L	L	U	U	U	U	L	L	L	L
Gattuso et al. [27]	L	L	U	U	U	U	L	L	L	L
Wang et al. [28]	L	L	U	U	U	U	L	L	L	H
Wang et al. [29]	L	L	U	U	U	U	L	L	L	H
Kolasa et al. [30]	L	L	U	U	U	U	L	L	L	H
Funk et al. [32]	L	L	U	U	H	U	L	L	L	H
García-Cabrerizo et al. [33]	L	L	L	U	U	U	L	L	L	L
Huang et al. [34]	L	L	U	U	U	U	L	L	L	H
Thomas et al. [35]	L	L	U	U	H	U	H	L	L	H
Liu et al. [36]	U	L	U	U	U	U	L	L	L	H
Zhao et al. [31]	L	L	U	U	L	U	L	L	L	H

Note: Q1, sequence generation; Q2, baseline characteristics; Q3, allocation concealment; Q4, random housing; Q5, blinding of caregivers/investigators; Q6, random outcome assessment; Q7, blinding of outcome assessors; Q8, incomplete outcome data; Q9, selective outcome reporting; Q10, other sources of bias. L, low risk; H, high risk; U, unclear risk.

**Table 5:** Summary of risk-of-bias assessments across SYRCLE domains for included animal experimental studies.

Domain	Low Risk *n* (%)	Unclear Risk *n* (%)	High Risk *n* (%)
Q1 Sequence generation	13 (86.7)	2 (13.3)	0
Q2 Baseline characteristics	15 (100)	0	0
Q3 Allocation concealment	1 (6.7)	14 (93.3)	0
Q4 Random housing	0	15 (100)	0
Q5 Blinding of caregivers/investigators	1 (6.7)	12 (80.0)	2 (13.3)
Q6 Random outcome assessment	0	15 (100)	0
Q7 Blinding of outcome assessors	13 (86.7)	1 (6.7)	1 (6.7)
Q8 Incomplete outcome data	15 (100)	0	0
Q9 Selective outcome reporting	15 (100)	0	0
Q10 Other sources of bias	4 (26.7)	0	11 (73.3)

## Data Availability

No new data were created or analyzed in this study. Data sharing is not applicable to this review article.

## Abbreviations

5-HT, 5-hydroxytryptamine; 5-HT_1__x001D, 5-HT_1_B receptor; 5-HT_2a_, 5-HT_2_A receptor; 5-HTT, serotonin transporter; AAV, adeno-associated virus; ALFF, amplitude of low-frequency fluctuations; AMG, amygdala; Arc, activity-regulated cytoskeleton-associated protein; AU, alcohol use; BDI, Beck Depression Inventory; BDI-1A, BDI-II Anxiety subscale; BEAQ, Brief Experiential Avoidance Questionnaire; BLA, basolateral amygdala; CB1R, cannabinoid receptor type 1; CEQ, Craving Experience Questionnaire; COMP, compound (psilocybin formulation); CORT, cortex; CREB, cAMP response element-binding protein; CeA, central amygdala; DAG, diacylglycerol; DMN, default mode network; DRN, dorsal raphe nucleus; EBI, Emotional Breakthrough Inventory; EGR1, early growth response 1; EN, executive network; EPM, elevated plus maze; ERK, extracellular signal-regulated kinase; fMRI, functional magnetic resonance imaging; FST, forced swim test; GABA, γ-aminobutyric acid; Glu, glutamate; GPCR, G-protein-coupled receptor; Gria1, glutamate ionotropic receptor AMPA type subunit 1; HADS, Hospital Anxiety and Depression Scale; HYP, hypothalamus; 5D-ASC, 11-Dimensional Altered States of Consciousness Scale; IP3, inositol trisphosphate; Ki-67, marker of proliferation Ki-67; LC, locus coeruleus; LSD, lysergic acid diethylamide; MADRS, Montgomery–Åsberg Depression Rating Scale; mPFC, medial prefrontal cortex; mTOR, mechanistic target of rapamycin; NAc, nucleus accumbens; NeuroD1, neurogenic differentiation 1; NORT, novel object recognition test; NT3, neurotrophin-3; NT4, neurotrophin-4; Ntrk2, neurotrophic receptor tyrosine kinase 2; OFC, orbitofrontal cortex; OFT, open field test; PAG, periaqueductal gray; PANAS, Positive and Negative Affect Schedule; PAP, preparation-assisted psychotherapy; PFC, prefrontal cortex; PKC, protein kinase C; PL, prelimbic cortex; PSD95, postsynaptic density protein 95; QIDS-SR, Quick Inventory of Depressive Symptomatology–Self-Report; QIDS-SR-14, QIDS-SR 14-item version; QIDS-SR-16, QIDS-SR 16-item version; QLES-Q-SF, Quality of Life Enjoyment and Satisfaction Questionnaire–Short Form; RCT, randomized controlled trial; SDS, Sheehan Disability Scale; SI, social interaction test; SN, salience network; SNRIs, serotonin–norepinephrine reuptake inhibitors; SPT, sucrose preference test; SSRI, selective serotonin reuptake inhibitor; STAI-T, State-Trait Anxiety Inventory–Trait; STG, superior temporal gyrus; TCAs, tricyclic antidepressants; TrkB, tropomyosin receptor kinase B; vmPFC, ventromedial prefrontal cortex; WSAS, Work and Social Adjustment Scale; WBSI, White Bear Suppression Inventory; WIS, Wistar rat; WKY, Wistar-Kyoto rat; 2-AG, 2-arachidonoylglycerol; MAOIs, monoamine oxidase inhibitors.
